# Supplementary material for: The central coupler of ClpXP controls intersubunit communication and couples intrasubunit chemical energy with force generation
Source: Nat Commun. 2026 Oct 12;17:11580. doi: 10.1038/s41467-026-78225-1 (PMC13752256; doi:10.1038/s41467-026-78225-1)
Supplement: Supplementary file 2 [file 41467_2026_78225_MOESM2_ESM.pdf]

nature portfolio

Peer Review File

The Central Coupler of the AAA+ ATPase ClpXP Controls Intersubunit Communication and Couples the Conversion of Chemical Energy into the Generation of Force

Corresponding Author: Professor Carlos Bustamante

This file contains all reviewer reports in order by version, followed by all author rebuttals in order by version.

Version 0:

Reviewer comments:

Reviewer #1

(Remarks to the Author)
The authors combined single molecule optical tweezers and CryoEM to study the role of the central couple in ClpX. They use a comprehensive set of mutants to dissect the role of the coupler in intersubunit communication and mechanochemical coupling. The study is novel and well written. The conclusions are sound based on the results provided. However, there are many parts of the manuscript were the methods are insufficiently described. Given the technical nature of single molecule optical tweezers experiments and analysis, the authors should provide more methodological differences. Also, there are some structural analysis that would benefit a broader readership, indicated below.

Major Comment: Step-Finding Algorithm - Insufficient description and validation of the step-finding algorithm

The description of the step-finding algorithm is limited to a citation of Ref. 9, with no additional details on its implementation. This lack of methodological clarity makes it difficult to reproduce the analysis or to properly assess the validity of the reported results. A more complete description and validation of the algorithm are essential. In particular, the authors should address the following points:

a. Control analyses and limits of detection.
What control analyses were performed to determine the lower limits of detectable step size and dwell time? For example, did the authors simulate trajectories containing heterogeneous step sizes and stochastic dwell times under different noise conditions, including noise levels comparable to those expected for the experimental translocation trajectories? Without such controls, it is unclear how the authors ensured that the algorithm is not overfitting noise or, conversely, underfitting true steps.

b. Criteria for step inclusion.
What is the minimum number of consecutive steps required for inclusion in the analysis? Clarification of these criteria is necessary to understand how the dataset was filtered and interpreted.

c. Force dependence of step analysis.
Were steps analyzed as a function of force, or were all steps pooled together regardless of force? Given that force changes as the motor translocates the unfolded polypeptide, ignoring force dependence could significantly affect step size and dwell time distributions.

d. Dataset size and statistics.
How many individual steps were included in the analysis, and how many trajectories were collected for the wild type and for each mutant? These numbers are critical for assessing the statistical robustness of the conclusions.

e. Use of alternative step-detection methods.
Did the authors apply any additional step-detection approaches? A step-finding algorithm will always return an output of steps and dwells, even in cases of overfitting. If the authors believe this is not the case here, they must explicitly justify why.

f. Independent, non-algorithmic validation.
The authors should complement the step-finding analysis with an independent, more statistical approach that does not rely on step-finding algorithms, such as pairwise distance distribution (PWD) analysis. Such methods can independently reveal characteristic step sizes, particularly for repetitive or consecutive steps, and would provide important validation of the results.

In summary, the manuscript requires a substantially more detailed description of how the step-finding algorithm was implemented, what controls were performed to assess overfitting and underfitting, and what additional analytical tools were used to validate the results. In its current form, the methodological description is insufficient for rigorous evaluation and reproducibility.

Other Important Comments:
1. The authors motivate their work by drawing on a previous study of the T4 DNA clamp loader, in which residue Q118 (Q208 in ClpX) is described as a “central coupler” that maintains structural rigidity. While the authors cite Ref. 20 to indicate that ClpX and other AAA+ motors share this architectural feature, the manuscript would benefit from a more explicit comparison between the central coupler in ClpX (Figure 1C) and that of the clamp loader. For example, how conserved are these structures quantitatively (e.g., RMSD)? Given that the substrates processed by the clamp loader and ClpX are fundamentally different, it is plausible that there are not only similarities but also important differences. Expanding this comparison would help the reader better appreciate the relevance and novelty of the present work.

2. Figure 1B is cited in line 53, prior to the introduction of several residues and motifs that are not labeled in the figure panel itself. This makes the text difficult to follow and forces the reader to repeatedly move between the text and the figure. For clarity, the authors should either label these motifs in the figure or clearly indicate in the text or figure legend which motifs are shown, and which are not.

3. Figure 1C is cited in line 64, but the accompanying text describes the clamp loader rather than ClpX. This issue is closely related to point 1 above. Again, a direct comparison between the clamp loader and ClpX would improve clarity and strengthen the conceptual framework of the manuscript.

4. In line 109, the Results section is labeled as Section 4, but it should be Section 3.

5. Figure S1 shows representative steps and is cited in line 108. As discussed in the first part of this review, the manuscript requires a more complete description of how these steps were detected, selected, and analyzed. In addition, appropriate control analyses should be presented to demonstrate that the step-finding procedure does not suffer from overfitting.

6. Figure 1D shows multiple configurations of ClpX mutants; however, some of these configurations (e.g., WMMWMM and WWMMWM) are not represented in the single-molecule optical tweezers experiments. If optical tweezers data were not collected for these mutants, the authors should explicitly state this and explain the rationale.

7. ClpX complexes containing five or six mutated subunits still show substrate degradation in gel-based assays, albeit at a much slower rate than other mutants. However, these same mutants do not show translocation of unfolded polypeptide in the optical tweezers experiments. Is this discrepancy due to extreme force sensitivity, or is another explanation possible?

8. The authors define a pause threshold of 1 s, but it is unclear how this value was chosen. The manuscript would benefit from a detailed explanation of this criterion, along with a histogram that clearly distinguishes dwell-time distributions from pause-time distributions.

9. The authors report an average step size of 2.2 nm for all mutants and for the wild type, while concluding that dwell times increase as the number of mutant subunits increases, with the exception of WMWMWM. Visual inspection of Supporting Figure 2 indeed suggests that step sizes are largely invariant across mutants. However, for the dwell-time distributions, the peak (or mode) appears very similar across all conditions. Long dwell events—such as those observed for WWMMMM—seem to be rare compared to events shared with the wild type or WWWWWWM. What fraction of dwells are longer in each mutant relative to wild type? The data suggest that the average dwell time may be dominated by a small number of long events rather than a systematic shift of the distribution. If so, this distinction should be clearly articulated. In addition, the choice of histogram bin widths for both step-size and dwell-time distributions is not described and should be explicitly stated.

10. The authors state that refolding of β-strand 11 of GFP occurs on a timescale of 230 ms. Does this value correspond to a lifetime? How was it determined? The authors should clarify whether this number was extracted from a histogram fitted to a single kinetic step and, if so, provide the relevant data and fitting details.

Reviewer #2

(Remarks to the Author)
Sosa 2026 review

Sosa et al. investigate the mechanism of coupling ATP hydrolysis to the mechanical tasks of polypeptide translocation and unfolding in the AAA-ATPase ClpX using a combination of biochemical, structural and single-molecule approaches. To systematically investigate inter-subunit coordination, the authors generate an array of 'single-chain' mutants in which the individual subunits of the hexamer are covalently linked, allowing defined placement of Q208A mutants in an otherwise wild-

type background. The mutant that eliminates H-bonds in the 'central coupler', a module that establishes cooperativity between neighboring subunits in ring-shaped AAA+ ATPases. Optical tweezers measurements were used to follow translocation of an unfolded protein in real-time, revealing instantaneous velocities that are similar for WT and for mutants containing at least 2 WT copies. In contrast, no no translocation is observed for versions with 1 or no WT copy. Michaelis-Menten-type analyses indicate that coupler mutants speed up ATP hydrolysis by increasing k_cat, resulting in increased ATP consumption per translocation step and, thus, weaker coupling between ATP hydrolysis and the mechanical step of polypeptide translocation. The presence of more than one mutant also reduces the ability of the motor to unfold GFP, suggesting that the mutants reduce the maximum force that ClpX is capable of generating. Indeed, the mutants reduce magnitude of externally applied force against which the motor can translocate at full velocity. Finally, structural analysis of ClpXP-substrate complexes with cryo-EM confirm the role of Q208 in H-bond mediated coupling. This analysis revealed a state with an extended pore-1 loop that had not previously been observed.

Overall, this beautifully combines several methodologies to provide a detailed picture of ClpX mechanochemistry. Collectivel, the results allow formulation of a model with four sequential stages of pore-1 loop movement during the ClpX power stroke, including a new pre-power stroke conformation. More broadly, it establishes how the 'central coupler' enables inter-subunit coordination and coupling of chemical and mechanical steps, with implications for the broad class of AAA+ motor proteins. I have only a few comments that the authors should address prior to publication.

- In the biochemical assay comapring various ClpX mutants (Fig. 2e), the MMWMMM and MMMMMM mutants show slow degradation, whereas these mutants are described as having no translocation activity in single-molecule measurements (Fig. 2d, and main text lines 117-119). What is the discrepancy due to? And can the results from the biochemical assays be analyzed quantitatively, e.g. by densitometry?

- Interestingly, the authors observe backward steps (Fig. 2f) in some recordings. This is somewhat surprising, given that the translocated peptide is degraded and slipping would thus be expected in dissociation - what is the molecular nature of these states? Also, the term 'backtracking', used here to describe backward steps, is commonly used in he context of RNA polymerase and might be misleading here. To avoid confusion, it might be better to use a different term (e.g. 'slipped'?).

- The WMWMWM mutant has by far the highest ATPase rate of any construct tested (Fig. S3), but the main text (lines 149-151) only mentions increased ATPase rates for MMMMMM and MMWMMM - why is that?

- The authors should check their references (ref. 29 lists only a title)

Reviewer #3

(Remarks to the Author)
Sosa et al. investigate how the conserved central coupler (three-helix module) in ClpX mediates inter subunit communication and mechanochemical coupling in ClpXP. The combination of optical tweezers, biochemical assays, and cryo-EM is compelling. However, several key conclusions require clarification and additional analysis, particularly regarding the distinction between substrate (degron) recognition and force generation, as well as the effect of the mutation on wild-type (WT) ClpX oligomerization (rather than the single-chain construct). The claim that the class II structures reported here represent a new conformation should also be more thoroughly analyzed in comparison with prior and recently determined structures. Below are my major and minor comments:

1. The proposed hydrogen-bond network involving Q208 (Fig. 1C) is not clearly defined. The authors should show the hydrogen-bond donors and acceptors and clarify whether these interactions involve side-chain or backbone atoms. Moreover, if the central claim is that Q208 uniquely maintains the integrity of this hydrogen-bond network, then mutations in other residues within the same network, provided they are not directly involved in ATP binding or hydrolysis, should produce comparable functional effects. Testing such residues would strengthen the conclusion that the observed phenotype arises from disruption of the hydrogen-bonded junction rather than from a Q208-specific effect.

2. Have the authors introduced the Q208A mutation into WT monomeric ClpX (as opposed to the single-chain construct) to assess its effect on ClpX oligomerization or stability?

3. The manuscript provides extensive single-molecule data; however, bulk kinetic parameters (KM and Vmax) for substrate processing are helpful to exclude the possibility that the Q208A mutations alter ssrA degron recognition. For example, mutations within the central coupler could loosen subunit packing or alter subunit arrangement, thereby reshaping the axial-channel conformational landscape relative to the wild type. Several prior studies (Mahmood A.S. et al., Cell Reports, 2022; Ghanbarpour, A. et al., Protein Science, 2025; Ghanbarpour, A. et al., Nature Communications, 2023) have shown that changes in ATPase hydrolysis or axial-channel conformation can affect substrate specificity and degron recognition. Without these kinetic measurements, it is difficult to conclude that the observed unfolding defects arise solely from impaired mechanochemical coupling rather than altered substrate recognition.

4. The claim that Class II represents a new substrate-engaged stalled conformation requires more rigorous comparison with recently reported ClpX/ClpXP structures, including quantitative RMSD analyses. The current comparisons appear largely

limited to Fei et al.'s ClpX structures, some of which were solved with Walker B mutations or with uncharacterized substrates. The authors should more clearly articulate how Class II differs structurally from previously described pre–power-stroke states (e.g., Ghanbarpour et al., Nature Communications, 2024). Additionally, how do the authors exclude the possibility that Class II reflects a mutation-stabilized off-pathway state rather than a physiologically relevant intermediate? It is notable that a comparable conformation has not been observed in wild-type ClpXP structures stalled with unfolding-resistant substrates (e.g., Ghanbarpour et al., Nature Communications, 2024). Addressing this discrepancy would be important to support the proposed mechanistic interpretation.

5. The authors should clarify whether the cryo-EM density is sufficient to confidently assign which portion of the substrate is engaged or being translocated. Given the mechanistic emphasis on an "engaged" or pre-power stroke state, it is important to determine whether the map resolution and density continuity allow assignment of specific substrate segments within the axial channel. If the density does not permit unambiguous modeling of the substrate, the conclusions regarding the precise engagement state should be appropriately toned down, as these particles could alternatively represent translocation or degradation complexes.

6. The Methods state that approximately one minute elapsed between the addition of ATPγS and grid freezing. Given the slow hydrolysis rate of ATPγS and the concentrations of ClpX, ClpP, and substrate used, it would be helpful for the authors to estimate the expected distribution of substrate-free, substrate-engaged, and translocation complexes under these conditions (e.g., Saunders et al., PNAS, 2020), and to assess whether the reported particle populations in each conformational state are consistent with the anticipated reaction conditions.

7. The cryo-EM data-processing method section should provide more complete reporting. Please include classification strategy, masking procedures, map sharpening parameters, and any non-default software settings. Q-scores, directional resolution plot, angular distributions, and sphericity scores should also be reported for each structure.

Minor comments:
1. Line 109: Section 3, not Section 4, etc.
2. Line 218: "WWWWM" one monomer designation appears to be missing.
Methods:
3. Section 1: "Nitrogen Liquid" should be "Liquid nitrogen."
4. Section 1: "0.44 µm filter" please verify whether this should be 0.45 µm?
5. Section 3: "1 m DTT" should be 1 mM DTT.
6. Section 5: "guanidium chloride" should be "guanidinium chloride."
7. Section 11: "preparated" should be corrected to "prepared."
8. "stored at -80oC" should be written as "stored at –80 °C."

Version 1:

Reviewer comments:

Reviewer #1

(Remarks to the Author)
The authors have successfully addressed all my comments. Their new manuscript is very strong and the quantitation and statistical analysis of the results is very convincing. This is a compelling story with broad relevance to the field of AAA+ motors. I have no additional comments.

Reviewer #2

(Remarks to the Author)
The authors have included additional analyses and descriptions that have substantially improved the manuscript, in my opinion. The concerns outlined in my review of the original manuscript have been fully addressed. In my reading, the revised manuscript also addresses the points raised by the other two reviewers, and the description of the results and interpretation is more rigorous and accessible now. This beautiful and timely study not only significantly advances our mechanistic understanding of the intricate ClpXP system, but provides much-needed quantitative understanding of the coupling and functional mechanisms of AAA+ motors more broadly.

Reviewer #3

(Remarks to the Author)
The authors have addressed most of my comments, and the manuscript has been substantially improved. However, there are still a few points that should be addressed before publication.

1. Supplementary Table S4 should be made more quantitative and clearly defined. The classifications “Away,” “Midway,” and “Closer” are not explained, making it difficult to understand how these assignments were made. The authors should specify the structural metric used for each comparison, including the atoms or residues measured, the structural alignment procedure, and the distance thresholds used for each classification. In particular, the column “Top subunit to polypeptide” is ambiguous, as a whole subunit does not have a unique distance to the substrate.

2. The new experiments examining the Q208A mutation in monomeric ClpX should be incorporated into the manuscript. While it is understandable that a given mutation may have different effects in the native enzyme and the covalently linked single-chain hexamer, the latter was specifically designed to distinguish defects in oligomerization from defects in mechanochemical function. The comparison between monomeric and single-chain Q208A is therefore directly relevant to the current study and strengthens the rationale for using the single-chain hexamer as the experimental system.

Open Access This Peer Review File is licensed under a Creative Commons Attribution 4.0 International License, which permits use, sharing, adaptation, distribution and reproduction in any medium or format, as long as you give appropriate credit to the original author(s) and the source, provide a link to the Creative Commons license, and indicate if changes were made.
In cases where reviewers are anonymous, credit should be given to 'Anonymous Referee' and the source.
The images or other third party material in this Peer Review File are included in the article’s Creative Commons license, unless indicated otherwise in a credit line to the material. If material is not included in the article’s Creative Commons license and your intended use is not permitted by statutory regulation or exceeds the permitted use, you will need to obtain permission directly from the copyright holder.
To view a copy of this license, visit https://creativecommons.org/licenses/by/4.0/

RESPONSE TO REVIEWERS' COMMENTS

Reviewer #1 (Remarks to the Author):

Major Comment: Step-Finding Algorithm - Insufficient description and validation of the step-finding algorithm

The description of the step-finding algorithm is limited to a citation of Ref. 9, with no additional details on its implementation. This lack of methodological clarity makes it difficult to reproduce the analysis or to properly assess the validity of the reported results. A more complete description and validation of the algorithm are essential. In particular, the authors should address the following points:

a. Control analyses and limits of detection.

What control analyses were performed to determine the lower limits of detectable step size and dwell time? For example, did the authors simulate trajectories containing heterogeneous step sizes and stochastic dwell times under different noise conditions, including noise levels comparable to those expected for the experimental translocation trajectories? Without such controls, it is unclear how the authors ensured that the algorithm is not overfitting noise or, conversely, underfitting true steps.

Answer. We thank the reviewer for raising this crucial point for the analysis of traces. To validate the Kalafut-Visscher step finding algorithm we performed simulations. The details of the simulations are now in the new section 10 of Materials and methods.

*"**10. Validation of the Kalafut-Visscher step finding algorithm.***

To validate the Kalafut-Visscher step finding algorithm, we generated simulated translocation traces of ClpXP, applied the algorithm and compared the ground-truth vs the found steps under different conditions (below, Supplementary Fig. 2, 3). The simulated traces were obtained with the following parameters:

- ***Step Size**: We tested both a 2 nm fixed step size (Supplementary Fig. 3c-d) and a 1, 2, and 3 nm equally distributed step size (Supplementary Fig. 3e-f), because we do not have the resolution to distinguish between these two possibilities.*
- ***Step Time**: Gamma distribution with shape factor 2. Two ATPs are burned per cycle with the release of two ADPs (one from the previous cycle after the power stroke and one for the resetting of the pore-1-loops in the present cycle), being ADP release rate-limiting [14]. Therefore, at least 2 rate-limiting events (shape factor 2) occur between every step. We used a mean dwell time of 0.2 sec to obtain the average experimental velocity of ~10 nm/s.*
- ***Sampling frequency**: 2.5kHz, which is the one we used in our data collection.*
- ***Noise**: The estimated noise of our data, by taking the standard deviation of (data – fit), is between 2.5 and 3.5 nm with the mode being ~3 nm, with some as large as 4.5 nm (Supplementary Fig. 3a). Therefore, in our simulations we tested the scenario of 3 nm (Supplementary Fig. 3c-f) and the worst-case scenario of 4.5 nm (Supplementary Fig. 4a-b).*

After obtaining the simulations, we applied the Kalafut-Visscher step finding algorithm with a penalty value of 2.5, because for most of the traces we used a value of 2.5 ± 0.2 for analysis. We also tested a penalty value of 2 and 3 to demonstrate the robustness of the algorithm (Supplementary Fig. 4c-d)."

We refer to this analysis in the main text as follows in lines 118 – 121 (changes are in red):

***"…**ClpXP translocation traces are made up of periods of dwells where the motor does not move and interrupted by translocation steps, which we determined using the Kalafut-Visscher step finding algorithm (see methods, supplementary Fig. 1). We validated the robustness of the algorithm with simulations (supplementary Fig. 2, 3)."*

We added to the supplementary information the new Supplementary Fig. 2 (below) to show noise in our traces, an example of a simulated trace, and the effect of the step size in the step finding algorithm.

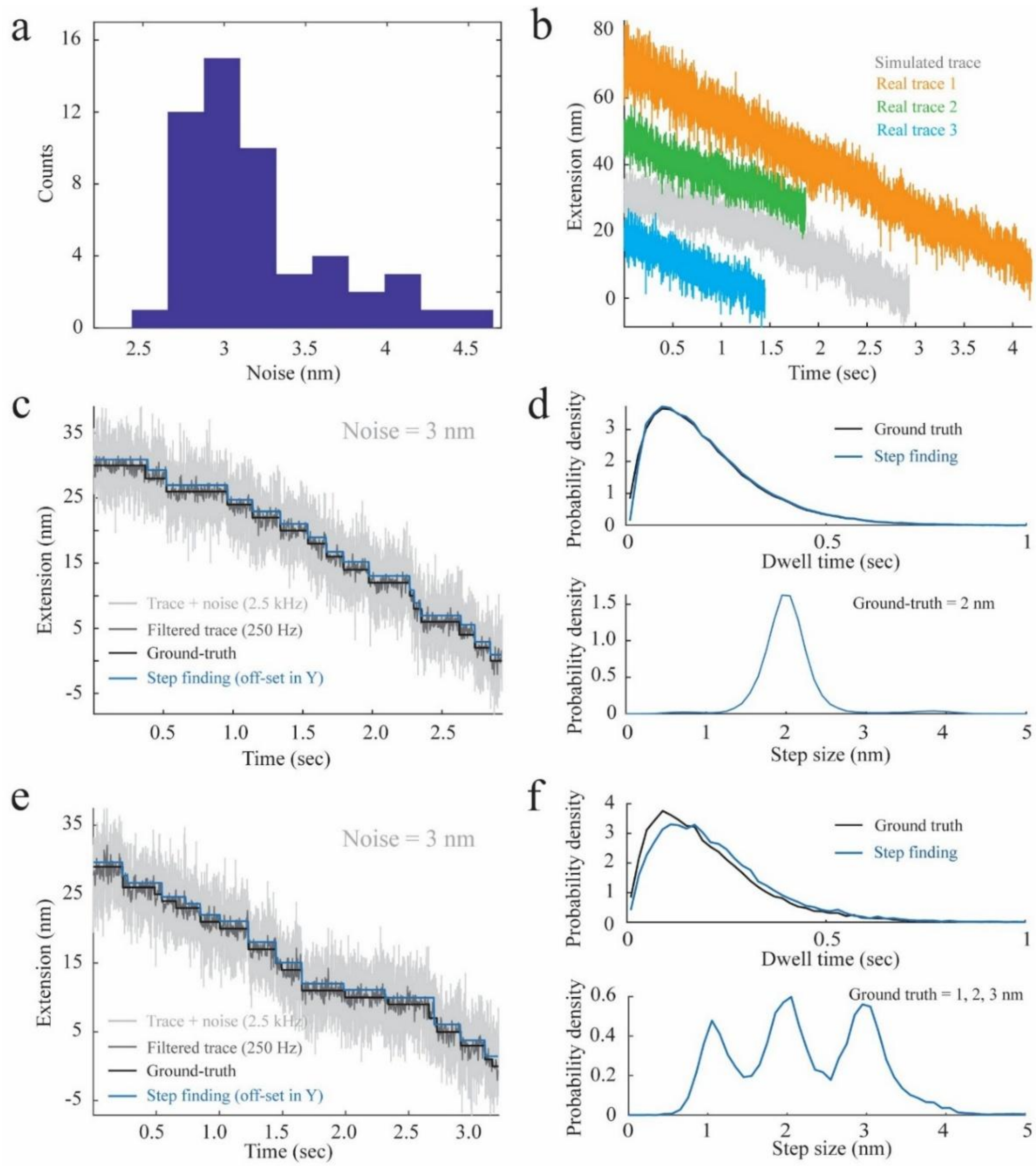

With the respective figure description.

“***Supplementary Figure 2. Validation of the step finding algorithm with simulations, effect of step size.*** *a) Noise distribution of the regions of polypeptide translocation without pauses and backtracking events from single molecule traces of the WWWWWW construct (n = 52 traces). b) Simulated trace (grey color) with the parameters of fixed step size = 2 nm and noise = 3 nm. For comparison, three examples of real traces are shown in different colors. c) Representative example of simulated trace at 2.5 kHz (light grey) and downsampled to 250 Hz (dark grey) for the case of step size = 2 nm and noise = 3 nm. The results of the step finding algorithm are shown in blue color (off-set in Y for clarity) vs the ground truth (black). d) Dwell time and step size distributions obtained with the step finding algorithm with a penalty value of 2.5 from simulated traces using a step size of 2 nm and noise of 3 nm. e) Representative example of simulated trace at 2.5 kHz (light grey) and downsampled to 250 Hz (dark grey) for the case of step size = 1, 2 and 3 nm and noise = 3 nm. The results of the step finding algorithm are shown in blue color (off-set in Y for clarity) vs the ground truth (black). f) Dwell time and step size distributions obtained with the step finding algorithm with a penalty value of 2.5 from simulated traces using and step size of 1, 2 and 3 nm and noise of 3 nm.*”

We added to the supplementary information the new Supplementary Figure 3 (below) to test the effect of the noise and penalty value on the step finding algorithm.

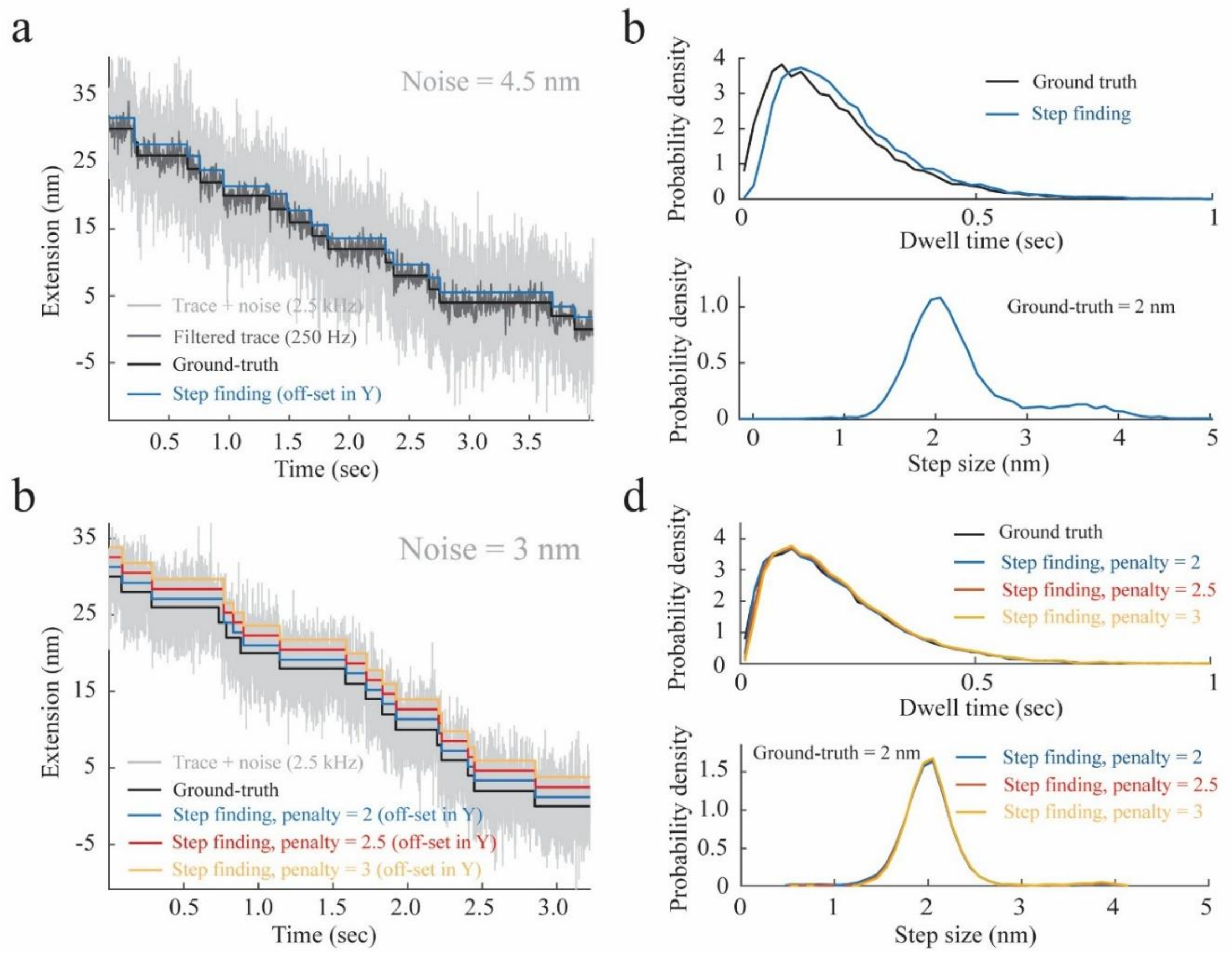

With the respective figure description.

"***Supplementary Figure 3. Validation of the step finding algorithm with simulations, effect of noise and penalty. a)*** *Representative example of simulated trace at 2.5 kHz (light grey) and downsampled to 250 Hz (dark grey) for the case of step size = 2 nm and noise = 4.5 nm. The results of the step finding algorithm are shown in blue color (off-set in Y for clarity) vs the ground truth (black).* ***b)*** *Dwell time and step size distributions obtained with the step finding algorithm with a penalty value of 2.5 from simulated traces using a step size of 2 nm and noise of 4.5 nm.* ***c)*** *Representative example of simulated trace at 2.5 kHz (light grey) and downsampled to 250 Hz (dark grey) for the case of step size = 2 nm and noise = 3 nm. The results of the step finding algorithm with penalty values of 2, 2.5 and 3 are shown in color blue, red and yellow, respectively (different off-set in Y for each fitting for clarity) vs the ground truth (black).* ***d)*** *Dwell time and step size distributions obtained with the step finding algorithm with a penalty value of 2, 2.5 and 3 from simulated traces using a step size of 2 nm and noise of 3 nm.*"

A paragraph discussing the validation of the simulation has now been added to the supplementary information

"***2. Validation of the step finding algorithm with simulations***

For the 2nm fixed step size, we see a great performance of the step finding algorithm because it gives the same result as the ground truth (Supplementary Fig. 2c), with only very rare events of missing steps (a very small fraction of 4 nm in the step size histogram of Supplementary Fig. 2d). In the variable step size case (Supplementary Fig. 2e), the step finding algorithm has some trouble identifying 1 nm steps (the triplet of peaks of 1, 2, and 3nm step sizes should be equal area, but the 1 nm one is smaller). This under representation leads to on average longer dwells due to these missed steps (Supplementary Fig. 2f). When we performed the simulations using a 4.5 nm noise (Supplementary Fig. 3a), the population of missed steps (4 nm) increases in the step size distribution histogram (Supplementary Fig. 3b), but still a good performance of the algorithm. Finally, we do not observe a significant difference in the dwell time and step size distribution if we use a penalty value of 2, 2.5 and 3 in the analysis of simulated traces with the step finding algorithm (Supplementary Fig. 3c-d)."

b. Criteria for step inclusion.

What is the minimum number of consecutive steps required for inclusion in the analysis? Clarification of these criteria is necessary to understand how the dataset was filtered and interpreted.

Answer: Details of analysis are now expanded in the new section 9 of materials and methods. For the specific case of steps, we wrote.

"*Below we provide the definition and details of analysis of each of the events in a single-molecule trajectory.*

Step. *In a single molecule trajectory in the optical tweezers experiments they are detected as shortening of the tether length by a distance between 1 – 6 nm during polypeptide translocation[14,16,17,46–49]. In this event, ClpX hydrolyzes ATP and releases the phosphate, triggering the power stroke of the motor by the*

motion of the ClpX pore loops[14,17]. For the case of the ClpX wild-type hexamer, the steps considered for analysis were the consecutive ones in 1 sec time frame, which correspond to ~6 steps assuming ~160 ms of an average dwell time (see definition below). For the case of ClpX hexamers harboring one or more mutant Q208A subunits, we considered traces displaying at least 6 steps (1 sec threshold is insufficient because mutant constructs have longer and more frequent pauses). Although unlikely, segments of single molecule trajectories containing less than 6 consecutive steps were not considered to determine step distribution and mean step value. In general, most of the steps for analysis came from more than 15 consecutive ones…."

c. Force dependence of step analysis.

Were steps analyzed as a function of force, or were all steps pooled together regardless of force? Given that force changes as the motor translocates the unfolded polypeptide, ignoring force dependence could significantly affect step size and dwell time distributions.

Answer: We did not pool together all the steps during polypeptide translocation in all the range of forces tested for each ClpX mutant when determining the step distribution. That would have been incorrect because at some specific force, the polypeptide translocation velocity of the motor changes and that could affect the observed step size (see Fig. 4h). Nonetheless, the mean value and distribution of step sizes were determined only in the range between 6 to 9.5 pN. In this interval of forces, the polypeptide translocation velocity is invariant for all mutants, with only a subtle decrease for the quadruple mutant MWWMMM above 8 pN.

To be more precise and avoid confusion we now added the following lines at the end to the new section 9 of materials and methods.

"For the analysis of all events mentioned above (steps, dwell, backtracking, pause and unfolding), we only used the single-molecule traces collected between ~6 to ~9.5 pN because, in that range, the polypeptide translocation velocity remained unchanged in all ClpX mutants. The instantaneous velocity and force sensitivity of the motor were determined over all the range of forces excluding dwells larger than 1 sec which are interpreted as pauses"

d. Dataset size and statistics.

How many individual steps were included in the analysis, and how many trajectories were collected for the wild type and for each mutant? These numbers are critical for assessing the statistical robustness of the conclusions.

Answer: Data number of total traces per condition and number of steps are indicated in the captions of Fig. 2. To make it clear we now added the word "dwells-steps" instead of "dwells" because each step determined properly has a dwell associated to it.

"*…WWWWWW (N = 59 traces), WWWWWM (N = 63), WWWWMM (N = 69), WWWMWM (N = 57), WWWMMM (N = 63), WMWMWM (N = 54), and WWMMMM (N = 47). **e)** SDS-PAGE gel degradation of TitinCM-ssrA by different Q208A mutants. **f)** Top, backtracking probability. Bottom, pause probability per*

*trace and per Q208A hexamer mutants. Error bars = SEM. **g)** Average step size (nm) and dwell time (ms) during translocation, excluding pauses and backtracking, for WWWWWW (2829 dwells-steps), WWWWWM (2973 dwells-steps), WWWWMM (3061 dwells-steps), WWWMWM (1991 dwells-steps), WWWMMM (2107 dwells-steps), WMWMWM (1330 dwells-steps), and WWMMMM (1556 dwells-steps). Error bars = SEM."*

e. Use of alternative step-detection methods.

Did the authors apply any additional step-detection approaches? A step-finding algorithm will always return an output of steps and dwells, even in cases of overfitting. If the authors believe this is not the case here, they must explicitly justify why.

Answer. We now performed the PWD analysis for all mutants tested in the optical tweezers for the polypeptide translocation section of each trace. See results below.

f. Independent, non-algorithmic validation.

The authors should complement the step-finding analysis with an independent, more statistical approach that does not rely on step-finding algorithms, such as pairwise distance distribution (PWD) analysis. Such methods can independently reveal characteristic step sizes, particularly for repetitive or consecutive steps, and would provide important validation of the results.

Answer. We now refer to the results of the PWD as a Figure in the supplementary material and in lines 158-160 of the main text (changes are in red):

"*…lengthening of the dwells (Fig. 2g, Supplementary Table 1). The pair-wise distribution analysis (see methods) confirms that ClpX performs translocation steps of ~2 nm (Supplementary Fig.4).*"

The methodology to analyze the PWD of single molecules traces is now presented as part of the new section 9 of materials and methods:

"*To have an independent measurement of translocation steps that do not rely on step-finding algorithms during polypeptide translocation, we performed the pair-wise distribution (PWD) analysis[16,50,51]. Because ClpXP of WWWWWW can also perform backtracking and pauses during polypeptide translocation, we selected only regions of the trace without those events with a size spanning at least 20 nm. We obtained the PWD by taking the autocorrelation of traces filtered by a moving average filter of 30 points or 83 Hz (for the case of the wild type). Distributions of individual traces were graded by their periodicity, by integrating the power spectrum over the frequency range ±10% of the average value of the peak distribution and only the top 30% were selected for averaging[50,51]. We also performed a similar analysis for WWWWWM (filtered to ~83 Hz) and WWWWMM (filtered to 50 Hz). For the other constructs (WWWMWM, WWWMMM, MWWMMM, and WMWMWM) the PWD analysis did not show the expected periodicity of around 2 nm because in those traces, backtracking and pauses events are more frequent.*"

The PWD analysis for all single molecule construct is now presented as the new Supplementary Fig 5 with the figure description.

"***Figure S5. Pair-wise distribution (PWD) analysis.*** *PWD analysis of section of the polypeptide translocation traces selected, from the raw data, that do not contain visible pauses nor backtracking events. Not all traces for a given construct used for the step finding algorithm fulfilled such conditions, because we selected only a minimum distance of translocated polypeptide of 20 nm (around 10 steps). The more mutant subunits are present in the ClpX hexamer construct, the less likely it is to find such sections, the easier it is to miss such events and include them in the PWD analysis, and consequently, the periodicity gets lost. Number of traces used per mutant are indicated in each PWD plot.*"

The new Supplementary Fig. 5:

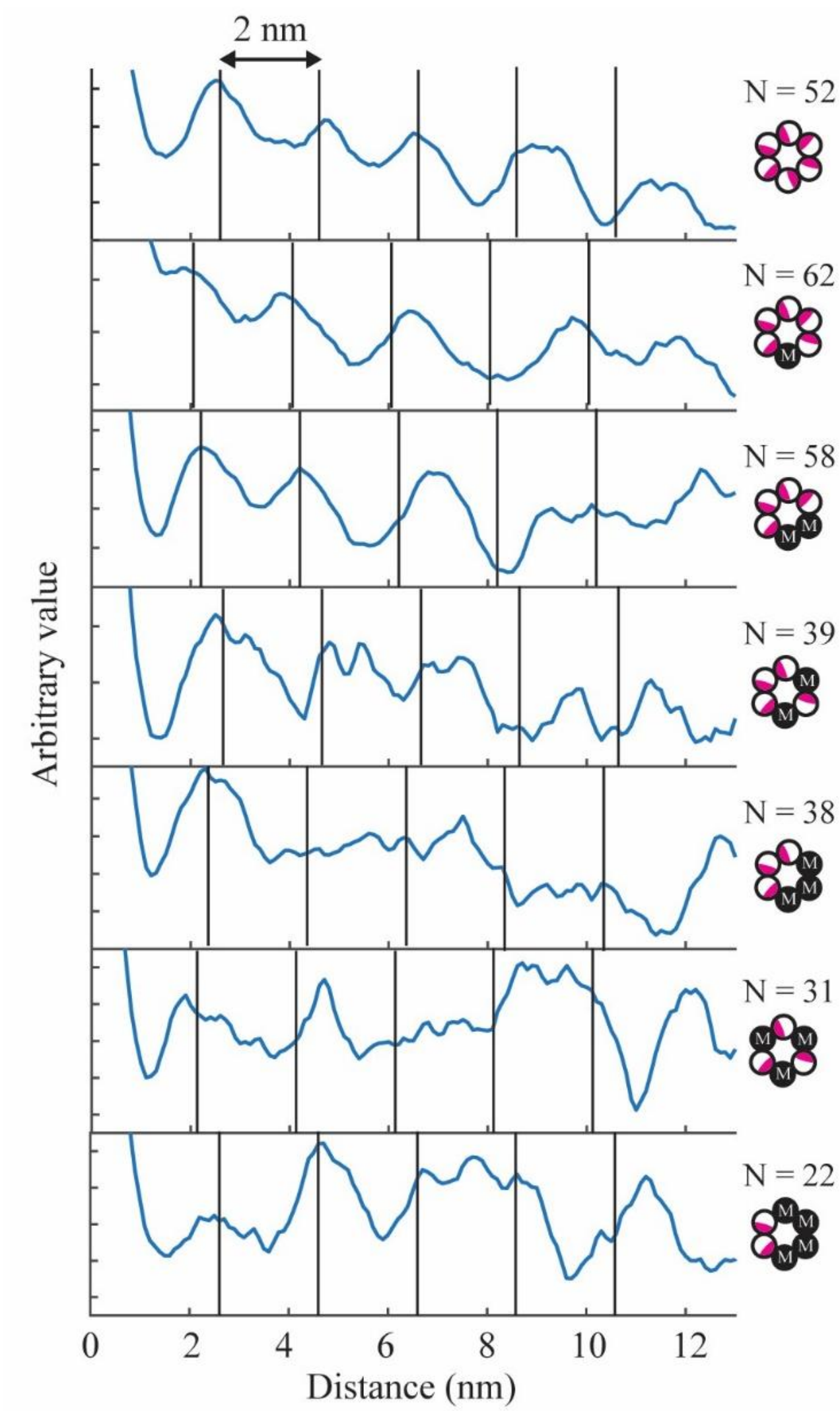

In summary, the manuscript requires a substantially more detailed description of how the step-finding algorithm was implemented, what controls were performed to assess overfitting and underfitting, and

what additional analytical tools were used to validate the results. In its current form, the methodological description is insufficient for rigorous evaluation and reproducibility.

Other Important Comments:

1. The authors motivate their work by drawing on a previous study of the T4 DNA clamp loader, in which residue Q118 (Q208 in ClpX) is described as a "central coupler" that maintains structural rigidity. While the authors cite Ref. 20 to indicate that ClpX and other AAA+ motors share this architectural feature, the manuscript would benefit from a more explicit comparison between the central coupler in ClpX (Figure 1C) and that of the clamp loader. For example, how conserved are these structures quantitatively (e.g., RMSD)? Given that the substrates processed by the clamp loader and ClpX are fundamentally different, it is plausible that there are not only similarities but also important differences. Expanding this comparison would help the reader better appreciate the relevance and novelty of the present work.

Answer. We thank the reviewer for the suggestion to highlight the similarity of and differences between the central couplers and connecting elements (nucleotide and walker-B) of ClpXP and the T4 DNA clamp loader. Fig. 1c has been modified to show the structural alignment of the central coupler and Walker-B motif of a subunit of the T4 DNA clamp loader (3U60), including its bound nucleotide, and the nucleotide of its adjacent subunit, relative to the same elements of *E. coli* ClpX (6PP7).

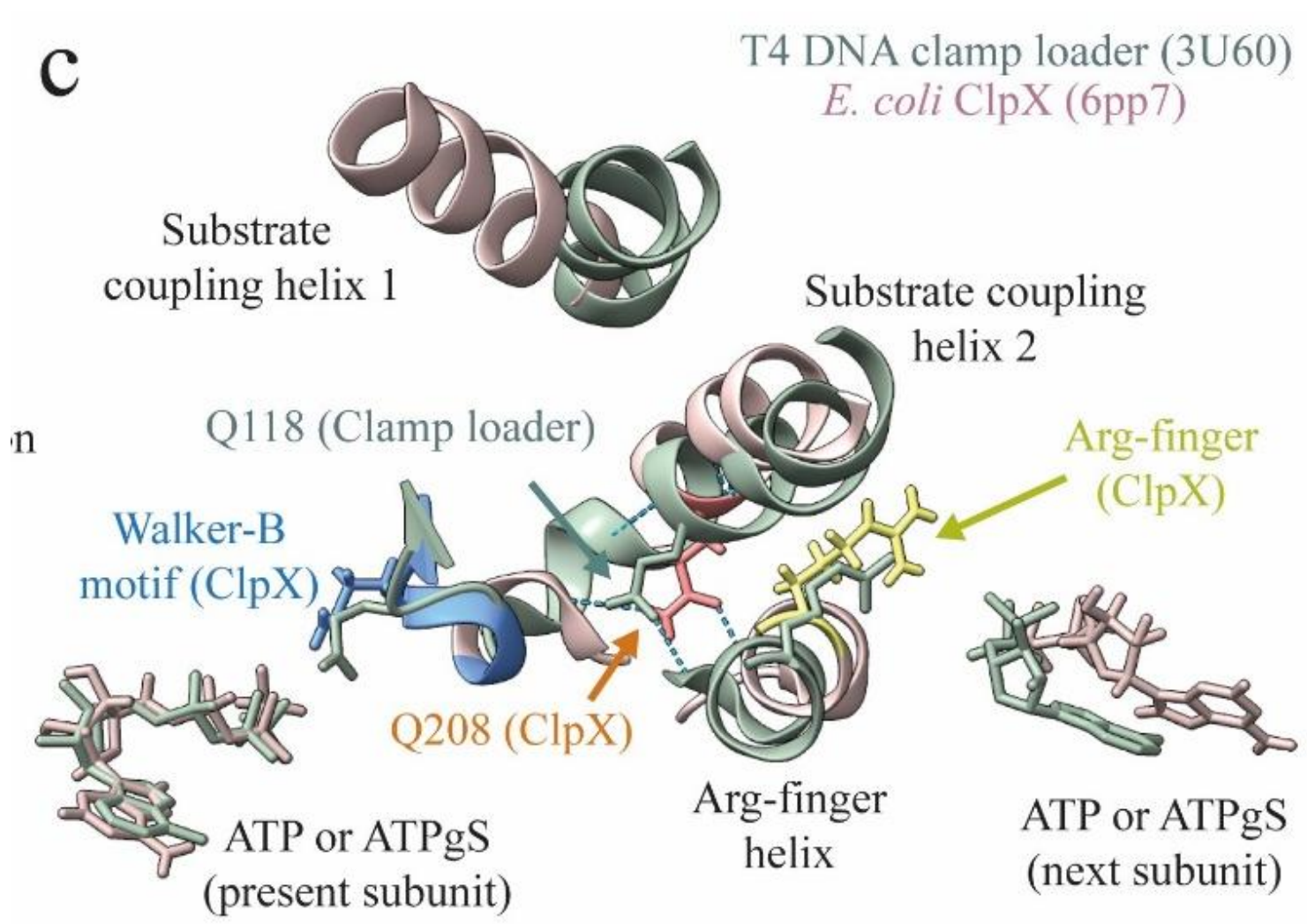

The new caption of Fig. 1c (changes are in red):

"***b)*** *ClpX hexamer (left), showing the general context of Glu208 within a ClpX subunit (right). The Arg-finger, Q208 and Walker-B of ClpX are shown in yellow, orange and blue, respectively.* ***c)*** *Comparing the structural alignment of the α-helices of the central coupler, the Walker-B, and the two ATP(γS) in contact with those elements of ClpX (6PP7 PDB, pink) with respect to those same elements in the T4 DNA clamp loader (3U60 PDB, calm green). The Arg-finger, Q208 and Walker-B of ClpX are shown in the same color code as in b. The Q118 of the T4 DNA clamp loader is indicated. H-bonds formed by Q118 and Q208 are shown as dotted lines…"*

Section 1 of the main text has been modified according to the new Fig. 1c to highlight the structural similarities and differences (changes are in red).

"1. The central coupler of the T4 DNA clamp loader and ClpX

The central coupler of the T4 DNA clamp loader is composed of three alpha helices: i) the Arg-finger helix, which contains the residue that senses the ATP status of the neighboring anti-clockwise subunit; ii) the substrate-coupling helix 2, where Q118 is located, which establishes the H-bonded network linking the catalytic Walker-B motif and the Arg-finger helix; and iii) the substrate-coupling helix 1 (Fig. 1c), which is connected to the DNA substrate[20]. Alignment of the central couplers, the Walker-Bs, the ATP(γS) nucleotides of a subunit and that of its clockwise neighbor of the T4 DNA clamp loader with the same elements of E. coli ClpX show that they share a similar architecture (RMSD = 1.1 Å). Importantly, the relevant glutamine Q208 of ClpX is structurally similar to Q118 of the clamp loader (Fig. 1c). However, the substrate-coupling helices 1 of both structures are the most divergent. This result is expected because these helices are connected to their different respective subtrates, dsDNA for the clamp loader and an unfolded polypeptide for ClpX."

2. Figure 1B is cited in line 53, prior to the introduction of several residues and motifs that are not labeled in the figure panel itself. This makes the text difficult to follow and forces the reader to repeatedly move between the text and the figure. For clarity, the authors should either label these motifs in the figure or clearly indicate in the text or figure legend which motifs are shown, and which are not.

Answer. We thank the reviewer for pointing this out. Now, to make it clear, in the introduction of the main text we only mention the Walker-B and the Arginine-finger motifs related to the **Figure 1b**. Other important motifs are mentioned as part of previous research to study their role in ClpX. Also, sensor-I and sensor-II are mentioned later in more detail when we present the cryo-EM structures. Lines 49-51 were modified as follows (changes are in red):

"*This cleft is lined with the Walker-B and Arginine-finger (Fig. 1b), which are involved in ATP hydrolysis and sensing of the neighboring nucleotide, respectively[4–7]. These motifs, also found in other AAA+ ATPases, including the Walker-B[8–10] and others like the IGF-loops involved in assembly with ClpP…*"

3. Figure 1C is cited in line 64, but the accompanying text describes the clamp loader rather than ClpX. This issue is closely related to point 1 above. Again, a direct comparison between the clamp loader and ClpX would improve clarity and strengthen the conceptual framework of the manuscript.

Answer. We have addressed this issue above.

4. In line 109, the Results section is labeled as Section 4, but it should be Section 3.

Answer: This typo is now corrected.

5. Figure S1 shows representative steps and is cited in line 108. As discussed in the first part of this review, the manuscript requires a more complete description of how these steps were detected,

selected, and analyzed. In addition, appropriate control analyses should be presented to demonstrate that the step-finding procedure does not suffer from overfitting.

Answer: This specific issue about the step-finding has been addressed above.

6. Figure 1D shows multiple configurations of ClpX mutants; however, some of these configurations (e.g., WMMWMM and WWMMWM) are not represented in the single-molecule optical tweezers experiments. If optical tweezers data were not collected for these mutants, the authors should explicitly state this and explain the rationale.

Answer. The two extra quadruple ClpX mutant configurations shown in the original figure 1d (see below left figure) contributed little to the understanding of the role of the central coupler and the mechanism of operation of ClpXP . Accordingly, we have removed their SDS-PAGE results for clarity, and we modified Fig. 1d, presenting only the set of mutants studied.

Original Figure

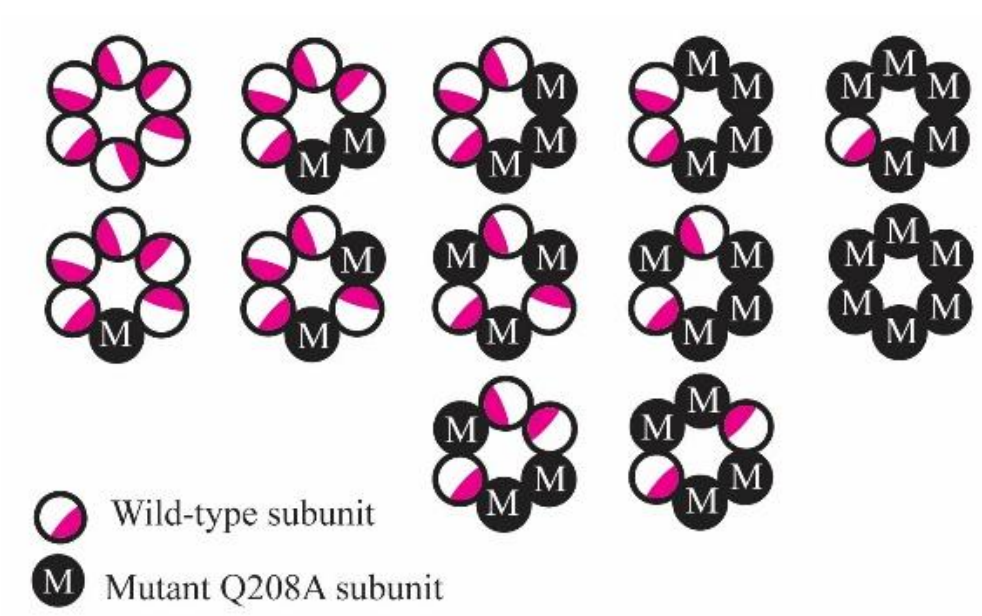

New Figure

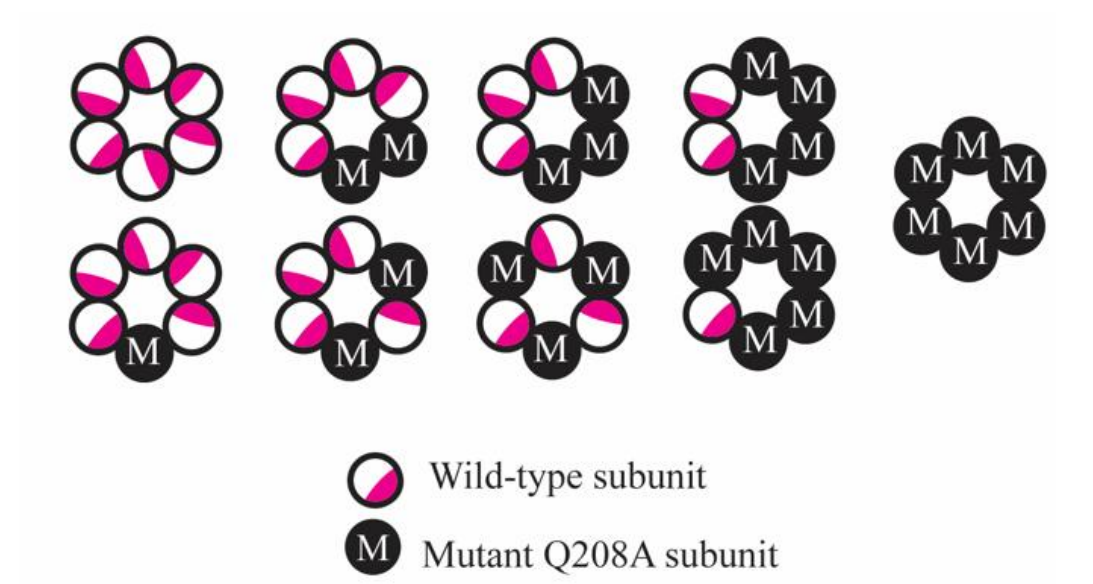

We added additional lines (92-95) at the end the section 1 of the main text to explain the rationale of the mutants we studied (changes are in red).

"…*Comparing the activity of WWWMMM vs WMWMWM provides the most contrasting effect of the configuration of mutant subunits. Additionally, WWWWWW, WWWMWM and WMWMWM reveal the effect of adjacent wild-type and mutant subunits relative to two adjacent mutant subunits (Fig. 1d)...*"

We also made the respective changes (shown in red) to the section 3 of the main text in lines 125-136:

"*…WWWWWM, WWWWMM, WWWMMM, and MWWMMM mutants displayed only a minor effect on their average instantaneous translocation velocity over unstructured polypeptides, compared to that of WWWWWW (Fig. 2c, d). A similar result is observed for other configurations of double (WWWMWM) and triple (WMWMWM) mutants (Fig. 2d). Also, MMWMMM and MMMMMM while able to engage with the ssrA-tag, as evidenced by the formation of a stable tether between the optical traps, they were unable to translocate, even when the opposing tension was lowered from ~6 to ~1 pN (Fig. 2d). The latter suggests that these mutants, with one or no wild-type subunits, are highly sensitive to force. The SDS-PAGE-based bulk degradation assay confirmed that WWWWWM, WWWWMM, WWWMMM, MWWMMM, and the mutants with a different configuration (WWWMWM and WMWMWM) have no*

impairment in their ability to degrade the Titin^{CM}-ssrA substrate while MMWMMM and MMMMMM also showed a dramatic effect on degradation (Fig. 2e, f)."

7. ClpX complexes containing five or six mutated subunits still show substrate degradation in gel-based assays, albeit at a much slower rate than other mutants. However, these same mutants do not show translocation of unfolded polypeptide in the optical tweezers experiments. Is this discrepancy due to extreme force sensitivity, or is another explanation possible?

Answer: Indeed, the reviewer is correct with the interpretation. Now the text in lines 129-136 has been modified as follows (Changes are in red):

"*…MMWMMM and MMMMMM while able to engage with the ssrA-tag, as evidenced by the formation of a stable tether between the optical traps, were unable to translocate, even when the opposing tension was lowered from ~6 to ~1 pN (Fig. 2d). The latter suggests that these mutants, with one or no wild-type subunits, are highly sensitive to force. The SDS-PAGE-based bulk degradation assay confirmed that WWWWWM, WWWWMM, WWWMMM,* ***,*** *MWWMMM, and the mutants with a different configuration (WWWMWM and WMWMWM) have no impairment in their ability to degrade the Titin^{CM}-ssrA substrate while MMWMMM and MMMMMM also showed a dramatic effect on degradation (Fig. 2e, f).*"

Also, we added the following text to lines 261 – 267:

"*Finally, these results also explain why MMWMMM and MMMMMM are not active in the optical tweezers. First, the more subunits harboring the Q208A mutation, the more sensitive is the ClpX hexamer to external forces. Second, although MWWMMM have a mild reduction in polypeptide translocation (in SDS-PAGE assay and by its activity below 8 pN in tweezers), F1/2 is reduced to ~9 pN. Since MMWMMM and MMMMMM display a dramatic effect on Titin^{CM}-ssrA degradation in the SDS-page assay (**Figure 2d**), it is not surprising that those ClpX hexamers will be highly sensitive to external force in the optical tweezers.*"

8. The authors define a pause threshold of 1 s, but it is unclear how this value was chosen. The manuscript would benefit from a detailed explanation of this criterion, along with a histogram that clearly distinguishes dwell-time distributions from pause-time distributions.

Answer: We thank the reviewer for raising this point. We address this issue in the new section 9 of materials and methods, where we now introduce the definition of a pause.

*"**Pause.** Up to now, there is not a rigorous definition of a "pause" in the context of ClpX during the translocation of an unfolded polypeptide. We also do not currently understand the biological role of a pause during protein degradation. Nonetheless, previous studies distinguished a pause from a dwell using an arbitrary threshold of 1 sec[14,16,17] or 2.5 sec[46,47]. Despite the arbitrariness of this value, it helped past single-molecule studies to find the number of ATPγS bound to ClpX to determine if one or more subunits are actively hydrolyzing ATP during a translocation step[14,17]. For consistency and comparative purposes, we consider a pause to be a dwell longer than 1 sec since we have used the same experimental conditions of those previous studies[14,16,17]…."*

9. The authors report an average step size of 2.2 nm for all mutants and for the wild type, while concluding that dwell times increase as the number of mutant subunits increases, with the exception of WMWMWM. Visual inspection of Supporting Figure 2 indeed suggests that step sizes are largely invariant across mutants. However, for the dwell-time distributions, the peak (or mode) appears very similar across all conditions. Long dwell events—such as those observed for WWMMMM—seem to be rare compared to events shared with the wild type or WWWWWWM. What fraction of dwells are longer in each mutant relative to wild type? The data suggest that the average dwell time may be dominated by a small number of long events rather than a systematic shift of the distribution. If so, this distinction should be clearly articulated. In addition, the choice of histogram bin widths for both step-size and dwell-time distributions is not described and should be explicitly stated.

Answer: The reviewer is right to point out that the dwell time distribution in Supplementary Fig. 2 (also including pauses longer than 1 sec) of the ClpX hexamer mutants look similar relative to that of the wild-type. To strengthen our claim that the dwell times of the mutants are indeed different although not dramatically so relative to the wild-type, we now performed the non-parametric Mann-Whitney U test of the dwell time distribution of each ClpX hexamer mutant vs wild-type and among them. The p-values of these comparisons are now in the new supplementary table S1. In sum, the dwell time distribution of all mutants is significantly different from that of the wild-type.

"***Supplementary Table 1:*** *p-values between dwell time distributions using the non-parametric Mann–Whitney U test*

	WWWWWM	***WWWWMM***	***WWWMMM***	***MWWMMM***	***WWWMWM***	***WMWMWM***
WWWWWW	*1.01E-07*	*9.35E-20*	*1.15E-61*	*1.05E-73*	*6.29E-56*	*2.65E-41*
WWWWWM		*7.38E-05*	*7.55E-33*	*1.35E-44*	*1.96E-28*	*6.25E-21*
WWWWMM			*3.58E-16*	*6.78E-26*	*1.07E-12*	*2.28E-09*
WWWMMM				*0.002534*	*0.307033**	*0.288828**
MWWMMM					*4.26E-05*	*0.000157*
WWWMWM						*0.915459**
WMWMWM						

**Square in orange are the p-values > 0.05, meaning there is no significant difference between those two distributions.*"

We refer to this new table in lines 157-158 (changes are in red)

"*…with the differences in velocity coming from lengthening of the dwells (Fig. 2g, Supplementary Table 1).*"

The reviewer also asked the question of the fraction of long dwells for each mutant. We measured this parameter as the pause probability for all ClpX constructs tested in the optical tweezers, which is mentioned in the text and plotted in Figure 2f top. However, as the reviewer pointed out above in question 8, it was not clear that the fraction of long dwells had been measured. Now, in the new section 9 of materials and methods, we write:

"…*We measured pause frequency by dividing the number of pauses over the total number of events in a polypeptide translocation region of a trace. If a trace does not have pauses, this value is equal to zero. Later, we calculate the pause probability (Fig. 2f) as the simple average of the pause frequency values of all traces for each ClpX hexamer mutant.*"

The choice of the bin for the distribution of steps is now explained in section 9 of materials and methods as follows:

"For plotting the step distribution, we used a bin size of 1 nm because this value is the shortest size of the step detected in our data and previous studies [14,16,17,46–49]."

The choice of the bin for the distribution of dwell times is now explained in section 9 of materials and methods as follows:

"For plotting the dwell time distribution, we used a bin size of 30 msec which is around twice the shortest dwell we detected in our data (~12 msec)."

10. The authors state that refolding of β-strand 11 of GFP occurs on a timescale of 230 ms. Does this value correspond to a lifetime? How was it determined? The authors should clarify whether this number was extracted from a histogram fitted to a single kinetic step and, if so, provide the relevant data and fitting details.

Answer. We thank the reviewer for pointing out the lack of clarity of the 230 ms value. Our laboratory has previously found that after the extraction of β11, it can refold back into GFP with a mean average refolding time constant of 230 ms. This value is the time constant from the exponential equation fitting to lifetime distribution of β11 at ATP concentration between 100 – 200 uM at opposing force of 7 – 9 pN. At ATP concentrations above 200 uM, those events are rarely seen (less than 1% in unfolding events for ClpX wild-type). In our study, we used saturating ATP conditions. Therefore, we cannot measure such lifetime from our data. However, we assume it is still true because we used the same experimental conditions.

To make it clear and avoid confusion we modified the main text in lines 219-224 as follows (changes are in red):

"*We previously determined[17] that the initial extraction of the C-terminal β-strand 11 (β11) of GFP represents the first mechanical barrier for successful unfolding (Fig. 4f). The mean refolding time constant of β11 into GFP was determined to be ~230 ms at low (< 200 μM) ATP concentrations[17]. Assuming this is true in our case, because we used similar experimental conditions with saturating ATP, if ClpX fails to translocate over the unfolded β11 in less than 230 msec, this element is likely to refold, and ClpX will have to start a new unfolding attempt.*"

We also modified the main text in lines 230-233 as follows (changes are in red) to make our results clearer.

"*Furthermore, for the case in which extraction of β11 leads to the full unfolding of GFP, the mean average time of this intermediate remains below 100 ms in WWWWWW, WWWWWM, and WWWWM. In contrast, this value increases to ~400 ms in WWWMMM (Fig. 4g).*"

Reviewer # 2

Sosa 2026 review

Sosa et al. investigate the mechanism of coupling ATP hydrolysis to the mechanical tasks of polypeptide translocation and unfolding in the AAA-ATPase ClpX using a combination of biochemical, structural and single-molecule approaches. To systematically investigate inter-subunit coordination, the authors generate an array of 'single-chain' mutants in which the individual subunits of the hexamer are covalently linked, allowing defined placement of Q208A mutants in an otherwise wild-type background. The mutant that eliminates H-bonds in the 'central coupler', a module that establishes cooperativity between neighboring subunits in ring-shaped AAA+ ATPases. Optical tweezers measurements were used to follow translocation of an unfolded protein in real-time, revealing instantaneous velocities that are similar for WT and for mutants containing at least 2 WT copies. In contrast, no no translocation is observed for versions with 1 or no WT copy. Michaelis-Menten-type analyses indicate that coupler mutants speed up ATP hydrolysis by increasing k_cat, resulting in increased ATP consumption per translocation step and, thus, weaker coupling between ATP hydrolysis and the mechanical step of polypeptide translocation. The presence of more than one mutant also reduces the ability of the motor to unfold GFP, suggesting that the mutants reduce the maximum force that ClpX is capable of generating. Indeed, the mutants reduce magnitude of externally applied force against which the motor can translocate at full velocity. Finally, structural analysis of ClpXP-substrate complexes with cryo-EM confirm the role of Q208 in H-bond mediated coupling. This analysis revealed a state with an extended pore-1 loop that had not previously been observed.

Overall, this beautifully combines several methodologies to provide a detailed picture of ClpX mechanochemistry. Collectivel, the results allow formulation of a model with four sequential stages of pore-1 loop movement during the ClpX power stroke, including a new pre-power stroke conformation. More broadly, it establishes how the 'central coupler' enables inter-subunit coordination and coupling of chemical and mechanical steps, with implications for the broad class of AAA+ motor proteins. I have only a few comments that the authors should address prior to publication.

- In the biochemical assay comparing various ClpX mutants (Fig. 2e), the MMWMMM and MMMMMM mutants show slow degradation, whereas these mutants are described as having no translocation activity in single-molecule measurements (Fig. 2d, and main text lines 117-119). What is the discrepancy due to? And can the results from the biochemical assays be analyzed quantitatively, e.g. by densitometry?

Answer. We thank the reviewer for pointing this out. Now the text in lines 129 – 136 has been modified as follows (Changes are in red):

"…MMWMMM and MMMMMM while able to engage with the ssrA-tag, as evidenced by the formation of a stable tether between the optical traps, were unable to translocate, even when the opposing tension was lowered from ~6 to ~1 pN (Fig. 2d). The latter suggests that these mutants, with one or no wild-type subunits, are highly sensitive to force. The SDS-PAGE-based bulk degradation assay confirmed that

WWWWWM, WWWWMM, WWWMMM, MWWMMM, and the mutants with a different configuration (WWWMWM and WMWMWM) have no impairment in their ability to degrade the Titin^{CM}-ssrA substrate while MMWMMM and MMMMMM also showed a dramatic effect on degradation (Fig. 2e, f)."

Likewise, we added the text in lines 261 – 267:

"*Finally, these results also explain why MMWMMM and MMMMMM are not active in the optical tweezers. First, the more subunits harboring the Q208A mutation, the more sensitive is the ClpX hexamer to external forces. Second, although MWWMMM have a mild reduction in polypeptide translocation (in SDS-PAGE assay and by its activity below 8 pN in tweezers), F1/2 is reduced to ~9 pN. Since MMWMMM and MMMMMM display a dramatic effect on Titin^{CM}-ssrA degradation in the SDS-page assay (Fig. 2d), it is not surprising that those ClpX hexamers will be highly sensitive to external force in the optical tweezers.*"

We also performed a densitometric analysis of the results of the SDS-PAGE degradation assay. We added this plot as the new Fig. 2f to provide quantitative support to our claim that the speed of degradation in solution (in the absence of force) barely changes from WWWWWW to MWWMMM but shows a dramatic decrease in the pentamutant (MWMMMM) and MMMMMM.

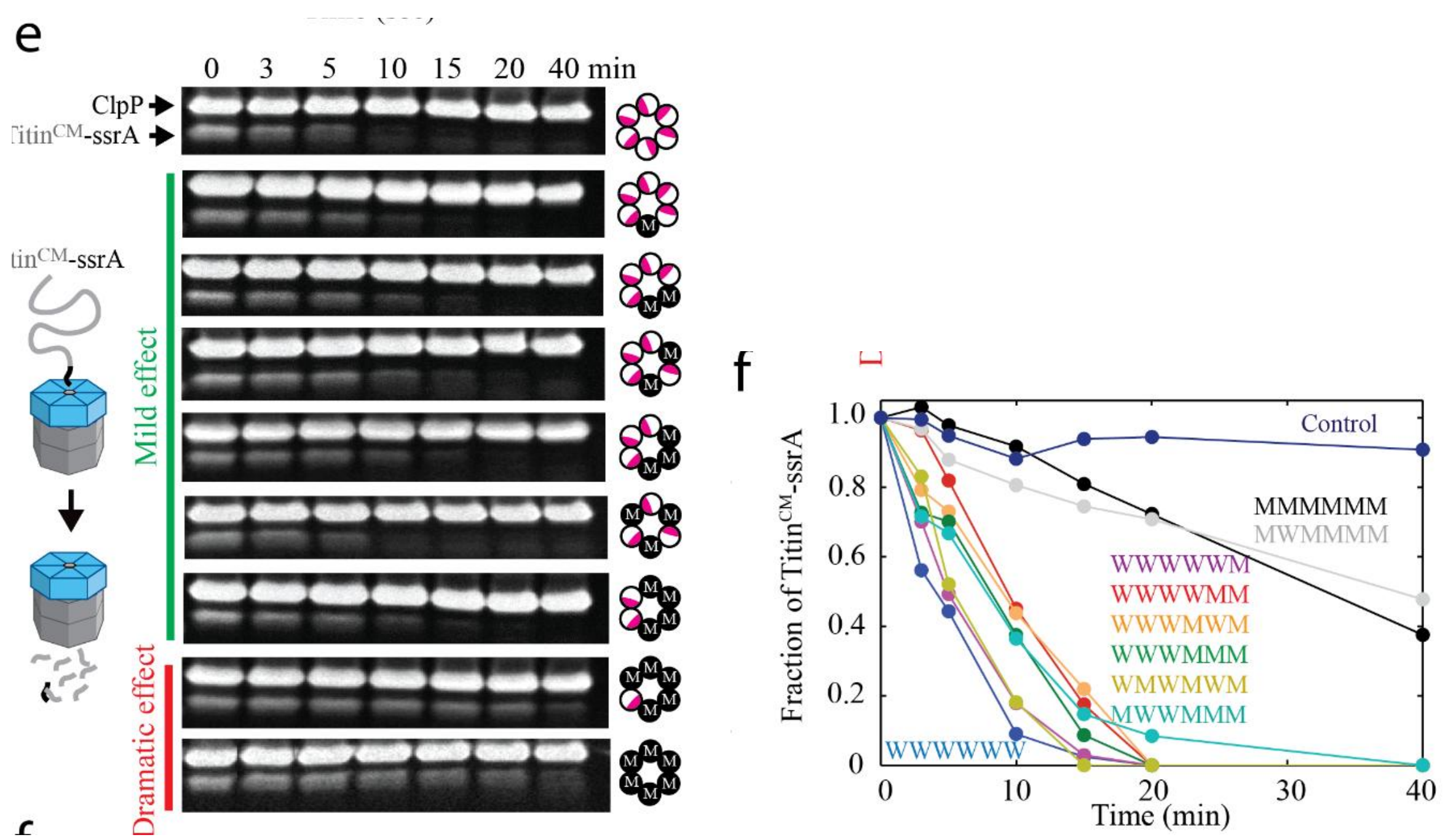

The caption of this **figure 2f** is:

"***f)** Plot of fraction of substrate remaining (Densiometric analysis) vs degradation time (min) of all constructs. Control is all the components of reaction but without ClpX.*"

- Interestingly, the authors observe backward steps (Fig. 2f) in some recordings. This is somewhat surprising, given that the translocated peptide is degraded and slipping would thus be expected in dissociation - what is the molecular nature of these states? Also, the term 'backtracking', used here to describe backward steps, is commonly used in he context of RNA polymerase and might be misleading here. To avoid confusion, it might be better to use a different term (e.g. 'slipped'?).

Answer: We thank the reviewer for raising this point. We realize that the term "backtracking" was not properly defined, although as shown in the Supplementary Fig. 4a these events are of comparable size to translocation steps. As such, these occurrences are very distinct from large backward event (>> 5 nm) sometimes observed. These are interpreted as slips, i.e., as resulting from polypeptide disengagement for a short period of time.

To clarify our use of the term "backtracking", we have modified the main text in lines 143 – 146 (changes are in red):

"*…Traces also displayed backward steps, which we refer to as "backtracking", because they are of comparable size to translocation steps (Fig. 2f, Supplementary Fig. 2a). However, they are much shorter than events of large tether increase (slips) that are likely due to temporary grip loss of the client polypeptide by ClpX.*"

A more detailed explanation is given in the new section 9 of materials and methods:

*"**Backtracking.** We found that during polypeptide translocation of single-molecule traces, the tether experienced, although unlikely, a lengthening ranging between 1 to 5 nm in a single event or in a stepwise manner, followed by normal translocation. Because they are of comparable size to translocation steps, we referred to them as backtracking. In contrast, events of large tether increase (>>5 nm) have a wide distribution of sizes and appear to be associated with a temporary loss of grip of ClpXP for its polypeptide substrate[16]. It is possible that the backtracking events represent short periods during which the motor can "walk backwards", pulling away the polypeptide chain from the ClpP chamber without disengagement. We quantified the backtracking frequency as the number of those events divided by the total number of forward and backward stepping events in a polypeptide translocation region of a trace. Then, the backtracking probability can be computed as the average of the backtracking frequency values of all traces for each ClpX hexamer mutant (Fig. 2f). Each backtracking step was considered individually even if they happened consecutively in a backtracking event."*

- The WMWMWM mutant has by far the highest ATPase rate of any construct tested (Fig. S3), but the main text (lines 149-151) only mentions increased ATPase rates for MMMMMM and MMWMMM - why is that?

Answer: In the original version of the paper, in the second paragraph of section 4 we only presented the results about MMMMMM and MMWMMM. For these constructs we attempted to obtain their corresponding Michaelis-Menten parameters, however Vmax could not be reached due to the large value of Km. Accordingly, for those mutants, we measured ATP binding using a modified nucleotide and found that the nucleotide dissociation constant remained unchanged. We note that in the third paragraph of section 4 of the same original version, we describe the analysis of the ATPase rates of all the other single (WWWWWM), double (WWWWMM, WWWMWM), triple (WWWMMM, WMWMWM) and quadruple (WWMMMM) mutants. In all those cases the Km values allowed us to reach Vmax.

To make the reading smoother and less confusing we changed the main text in lines 168 – 184 (changes are in red) as follows:

"*The ATPase assay of all hexamer mutants tested in the optical tweezers (Fig. 3) revealed that WWWWWW and WWWWWM have similar values of k_{cat}, that WWWWMM, WWWMWM, WWWMMM, and WWMMMM have ~1.5-fold larger k_{cat} values than WWWWWW, and that the triple variant WMWMWM has the highest k_{cat} value, ~4-fold higher (Fig. 3a). The K_m followed a similar trend (Fig. 3b). A linear relationship between K_m and k_{cat} is to be expected if Q208A does not change the nucleotide binding constant (K_D) but increases k_{cat}, according to the equation $K_m = K_D + (1/k_{on})k_{cat}$. Indeed, in most cases K_m and k_{cat} are linearly related, confirming that the loss of the H-bonded junction by the incorporation of Q208A raises the Km by increasing the corresponding k_{cat} of ATP hydrolysis, without affecting ATP binding (Fig. 3c). The only outlier in Figure 3C is the pentamutant, whose Km and kcat cannot be estimated reliably since it is not possible to reach saturation in the ATPase assay (Fig. 3a). The Km was much higher in the case of MMMMMM (Supplementary Fig. 3a). To have a further confirmation that K_D is also invariant in this case, we measured ATP binding via a fluorescence enhancement assay (see methods[24]) and found that WWWWWW and MMMMMM have identical ATP binding curves (Supplementary Fig. 3a). Thus, Q208A increases the K_m, via its effect on the k_{cat} without modifying the dissociation constant of the nucleotide. Taken together, these results indicate that Q208 in ClpX down regulates the ATP hydrolysis rate of the hexamer.*"

Reviewer #3 (Remarks to the Author):

Sosa et al. investigate how the conserved central coupler (three-helix module) in ClpX mediates inter subunit communication and mechanochemical coupling in ClpXP. The combination of optical tweezers, biochemical assays, and cryo-EM is compelling. However, several key conclusions require clarification and additional analysis, particularly regarding the distinction between substrate (degron) recognition and force generation, as well as the effect of the mutation on wild-type (WT) ClpX oligomerization (rather than the single-chain construct). The claim that the class II structures reported here represent a new conformation should also be more thoroughly analyzed in comparison with prior and recently determined structures. Below are my major and minor comments:

1. The proposed hydrogen-bond network involving Q208 (Fig. 1C) is not clearly defined. The authors should show the hydrogen-bond donors and acceptors and clarify whether these interactions involve side-chain or backbone atoms. Moreover, if the central claim is that Q208 uniquely maintains the integrity of this hydrogen-bond network, then mutations in other residues within the same network, provided they are not directly involved in ATP binding or hydrolysis, should produce comparable functional effects. Testing such residues would strengthen the conclusion that the observed phenotype arises from disruption of the hydrogen-bonded junction rather than from a Q208-specific effect.

Answer. Our claim is not that Q208 uniquely maintains the integrity of the hydrogen-bond network. Instead, Q208A forms H-bonds that may be part of all the H-bonds and other types of interactions, that maintain the rigidity of the central coupler. For the T4 DNA clamp loader, Subramanian *et al*., 2021 performed mutational sensitivity of each aminoacid, finding that Q118 (Q208 in ClpX) is the only residue, whose mutation, that cannot form H-bonds, hinders the activity of the T4 DNA clamp loader. As said above, for ClpX other residues may contribute to the H-bond network of its central coupler. However, as shown now in the new Figure 1c, Q118 in the clamp loader connects the Walke B with the Arginine finger helix, just as Q208 does in ClpX. Thus, while a mutational study of ClpX similar to what has been done by Subramanian for the clamp loader is out of the scope of the present work, our study has been directed to characterize the phenotypic effects of a mutation that alters in ClpX the same interactions that maintain the rigidity of the central coupler in the clamp loader. In response to this comment, we have now modified the text as follows (changes are in red) in lines 95 – 97 1 to make this point clear:

"*Among other possible residues forming H-bonds in the central coupler of ClpX, Q208A disrupts the network of H-bonds (Fig. 1c), weakening its interaction with the Walker-B and Arg-finger helix, resulting in a flexible central coupler*."

The new Fig. 1c is:

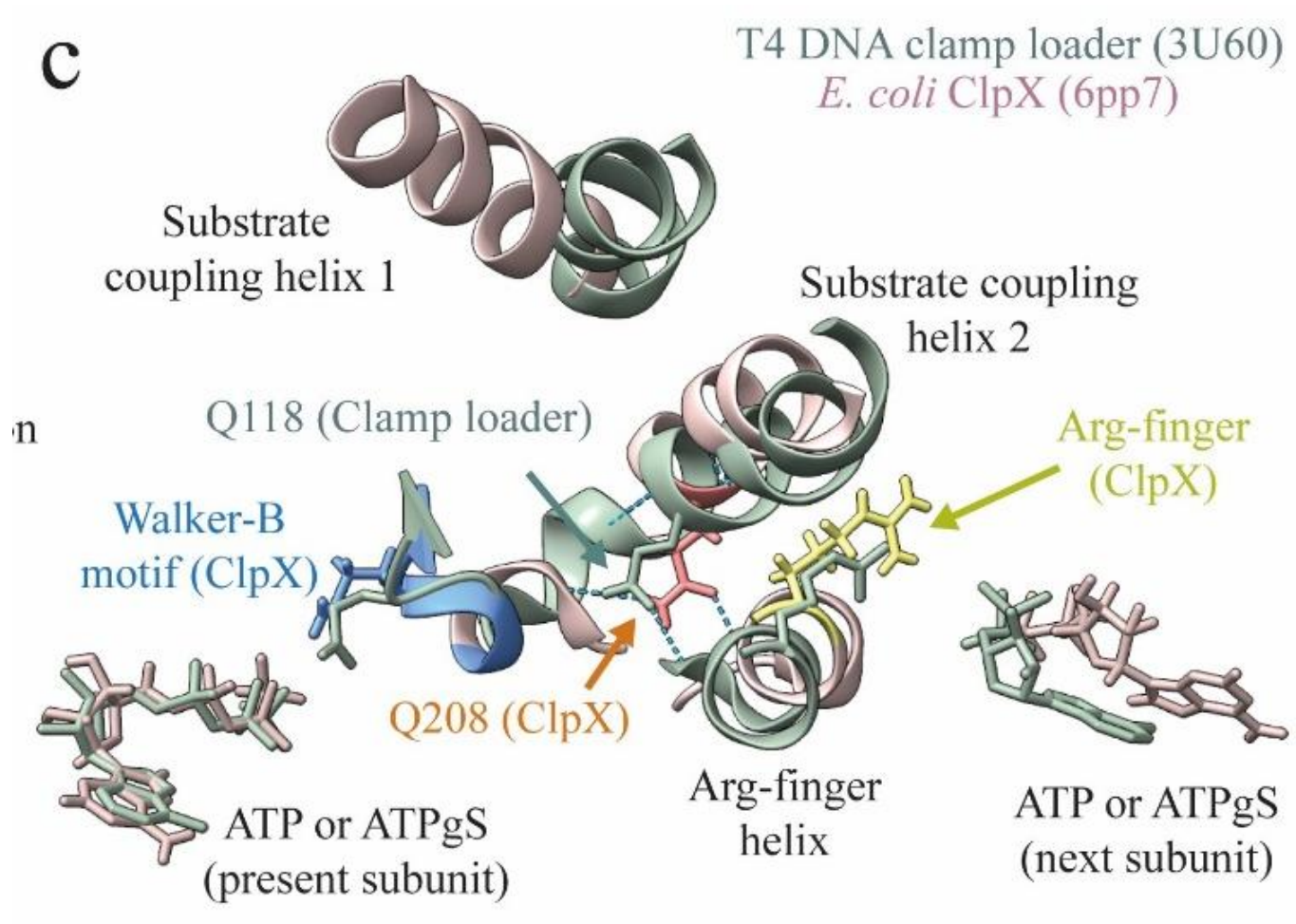

Now, the new caption of the Fig. 1c reads as follows

"…*The Arg-finger, Q208 and Walker-B of ClpX are shown in yellow, orange and blue, respectively.* ***c)*** *Comparing the structural alignment of the α-helices of the central coupler, the Walker-B, and the two ATP(γS) in contact with those elements of ClpX (6PP7 PDB, pink) with respect to those same elements in the T4 DNA clamp loader (3U60 PDB, calm green). The Arg-finger, Q208 and Walker-B of ClpX are shown in the same color code as in b. The Q118 of the T4 DNA clamp loader is indicated. H-bonds formed by Q118 and Q208 are shown as dotted lines…"*

2. Have the authors introduced the Q208A mutation into WT monomeric ClpX (as opposed to the single-chain construct) to assess its effect on ClpX oligomerization or stability?

Answer. We thank the reviewer for asking this interesting question. Yes, we investigated whether ClpX monomers carrying the Q208A mutation ($mono^{QA}$) could self-assemble into active hexamers or if these are formed only when linked into a single chain during the protein purification. As expected, wild-type monomers ($mono^{WT}$) exhibited a broad peak in the size exclusion chromatogram, with the earliest region corresponding to the hexamer form (Fig. A, see below). The presence of regions of lower molecular mass indicates that hexamers coexist at equilibrium with other species of lower oligomerization states. In contrast, the chromatogram of $mono^{QA}$ showed no absorbance around the hexamer peak, but peaks corresponding to lower oligomers (Fig. A).

In the case of single-chain constructs, however, the MMMMMM displayed a similar peak as WWWWWW in the size exclusion chromatogram indicating the presence of the hexamer form (Fig. A). Consistent with this observation, a ClpX degradation assay of $Titin^{CM}$-ssrA using SDS-page revealed that hexamers formed by $mono^{WT}$ and the single chain WWWWWW were fully active, that MMMMMM was active but significantly slower, and that $mono^{QA}$ showed no activity (Fig. A, inset). Strikingly, in the chromatogram of the Q208A monomer, we found two peaks of small molecules with absorbance at 260 nm (one of the peaks coincide with ATP, see Fig. A). This result suggests that $mono^{QA}$ can bind and retain bound nucleotides for a considerable amount of time to co-purify with them during size exclusion chromatography. Interestingly, the lower oligomerization species formed with $mono^{QA}$ showed zero ATPase activity.

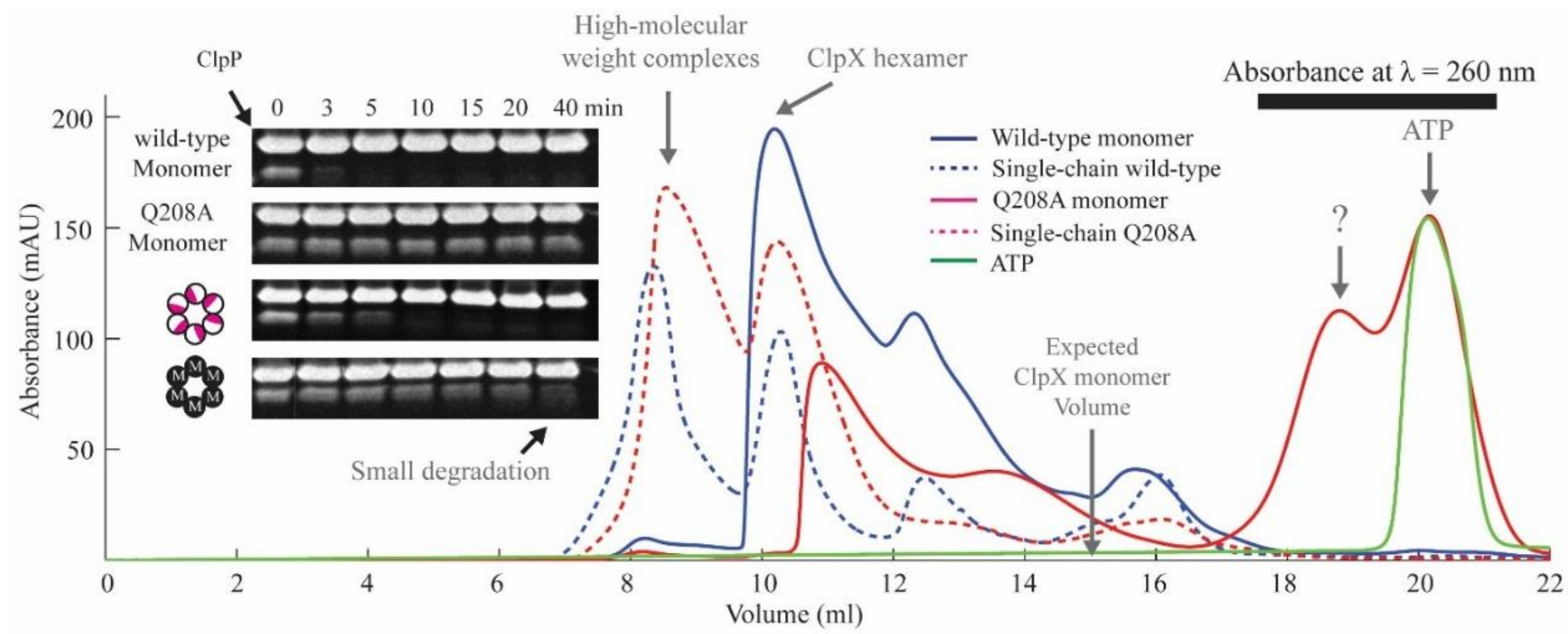

Figure A. Size exclusion Chromatogram of wild-type, Q208A ClpX monomers, WWWWWW and full Q208A hexamer mutant, MMMMMMM. Inset: SDS-PAGE gel of titin^{CM}-ssrA degradation by ClpXP in an interval of 40 min. The two peaks at ~19 ml and ~20 ml both have absorbance at 260 nm, and the latter coincided with free ATP.

We plan to pursue these studies and eventually publish these results within a more comprehensive study including a structural analysis using cryo-EM and/or native mass spectrometry.

3. The manuscript provides extensive single-molecule data; however, bulk kinetic parameters (KM and Vmax) for substrate processing are helpful to exclude the possibility that the Q208A mutations alter ssrA degron recognition. For example, mutations within the central coupler could loosen subunit packing or alter subunit arrangement, thereby reshaping the axial-channel conformational landscape relative to the wild type. Several prior studies (Mahmood A.S. et al., Cell Reports, 2022; Ghanbarpour, A. et al., Protein Science, 2025; Ghanbarpour, A. et al., Nature Communications, 2023) have shown that changes in ATPase hydrolysis or axial-channel conformation can affect substrate specificity and degron recognition. Without these kinetic measurements, it is difficult to conclude that the observed unfolding defects arise solely from impaired mechanochemical coupling rather than altered substrate recognition.

Answer. We thank the reviewer for raising this interesting point. Indeed, we believe that in the case of single-chain constructs with five or six mutant subunits, MWMMMM and MMMMMM, respectively, there could be an effect on substrate recognition. This is the case because the activity in solution (degradation of unfolded titin with the ssrA tag [titin^{CM}-ssrA] in the absence of force) is significantly reduced for these constructs. However, for wild-type, single, double, triple and quadruple mutants, the titin^{CM}-ssrA degradation activity is unaffected (see SDS-PAGE results in Fig. 2e). To support this conclusion quantitatively, we performed a densitometric analysis of the results of the SDS-PAGE degradation assay and presented in the new Fig. 2f (see below).

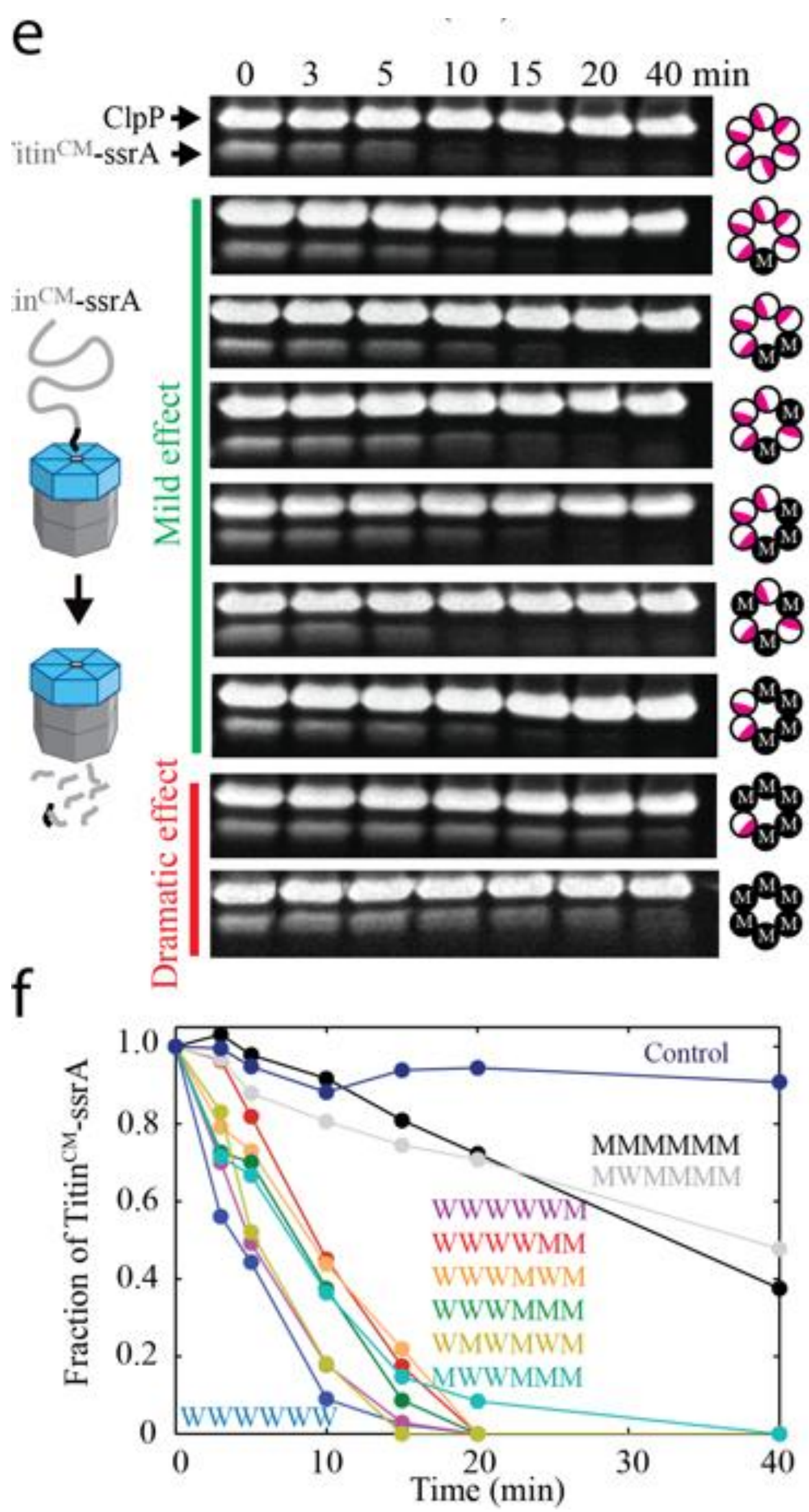

As seen in the figure, single, double, triple and quadruple mutants display little change in the speed of degradation of titin^{CM}-ssrA, indicating that any recognition effect if present is minimal. In contrast, the effect is very dramatic for the quintuple and full mutant. Since the substrate is already unfolded and activity is the same, we conclude that between substrate recognition and polypeptide translocation, the latter is still rate-limiting for most of the construct on which our present work is based. To make it clear, we are now only presenting the results of the SDS-PAGE assay for the mutants we tested in the optical tweezers and in the ATPase assay (the SDS-Page assay of other constructs was also confusing for another reviewer). See the modified Fig. 2e above.

Moreover, the velocity of polypeptide translocation of single, double, triple and quadruple mutants in the optical tweezers at forces below 9 pN, also displayed little change relative to the wild-type. These results are consistent with our findings of the SDS-PAGE assay.

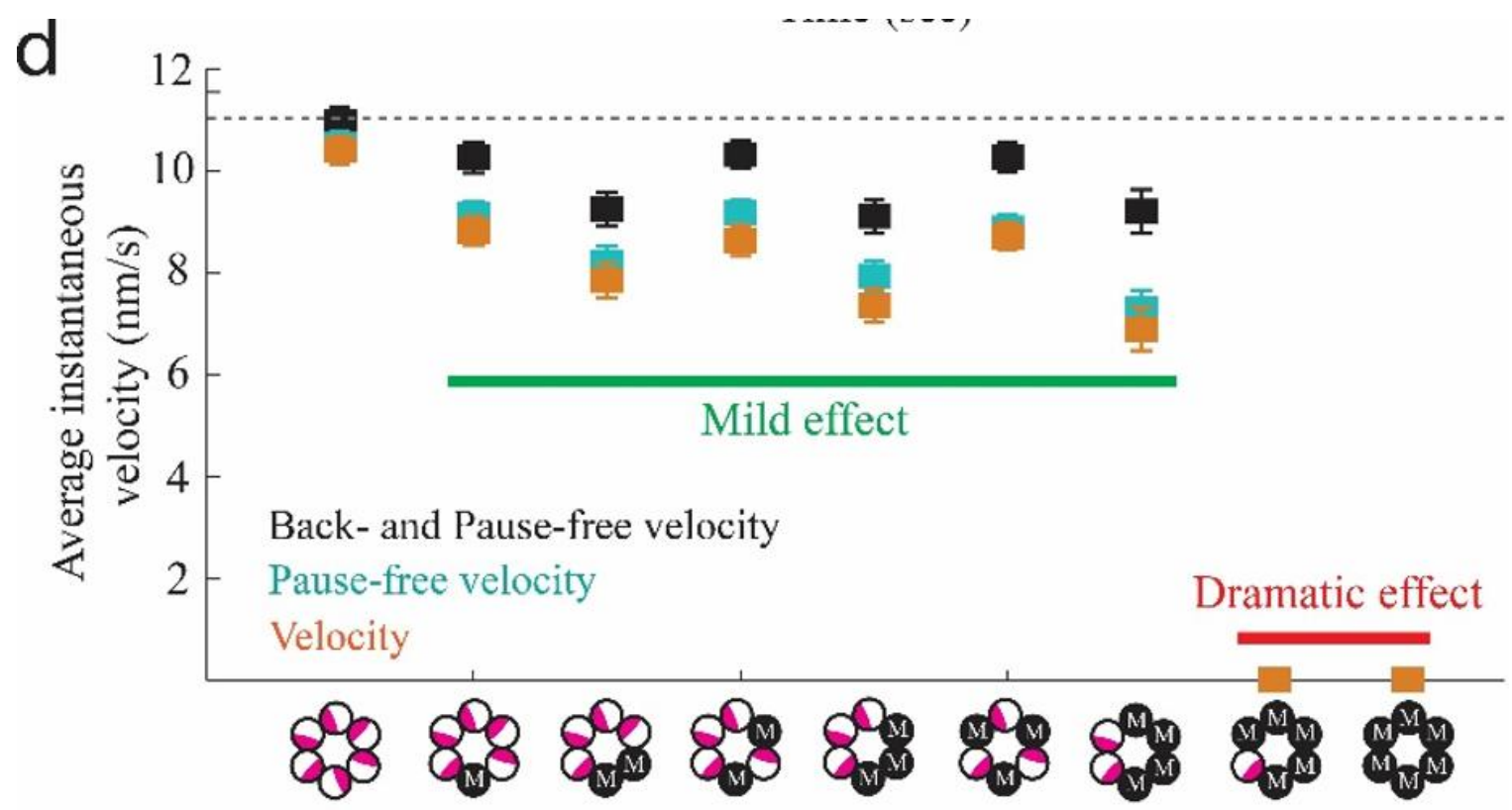

We are putting together all these points clearer in section 3 of the main text in lines 125 – 141 (changes are in red)

"*…WWWWWM, WWWWMM, WWWMMM, and MWWMMM mutants displayed only a minor effect on their average instantaneous translocation velocity over unstructured polypeptides, compared to that of WWWWWW (Fig. 2c, d). A similar result is observed for other configurations of double (WWWMWM) and triple (WMWMWM) mutants (Fig. 2d). Also, MMWMMM and MMMMMM while able to engage with the ssrA-tag, as evidenced by the formation of a stable tether between the optical traps, they were unable to translocate, even when the opposing tension was lowered from ~6 to ~1 pN (Fig. 2d). The latter suggests that these mutants, with one or no wild-type subunits, are highly sensitive to force. The SDS-PAGE-based bulk degradation assay confirmed that WWWWWM, WWWWMM, WWWMMM*, *MWWMMM, and the mutants with a different configuration (WWWMWM and WMWMWM) have no impairment in their ability to degrade the Titin^{CM}-ssrA substrate while MMWMMM and MMMMMM also showed a dramatic effect on degradation (Fig. 2e, f). Overall, a minimum of two subunits having a rigid central coupler, independent of their location within the hexamer, appears to be sufficient for near-optimal processive translocation of an unfolded polypeptide. Finally, the above single molecule and bulk results also indicate polypeptide translocation rather than substrate recognition is the rate-limiting step in the activity of ClpXP when the client protein is unfolded (Titin^{CM}-ssrA), a result consistent with previous bulk studies*[22]."

Furthermore, the effect on ClpX activity arises when the motor must overcome a high mechanical barrier like protein unfolding or high opposing force, as monitored in the optical tweezers. Note however, that in these experiments, the unfolded polypeptide substrate is already threaded within the ClpX pore and inside the ClpP proteolytic chamber such that the motor is stalled in front of GFP. Therefore, here again, substrate recognition plays no role in the inability of ClpX to unfold GFP in our optical tweezers assay. We clarify this point in lines 113 – 115 of the main text (changes are in red):

"*…Upon encountering GFP (the polypeptide chain is threaded from the top of the ClpX pore to the proteolytic chamber of ClpP), ClpXP pauses as it attempts to unfold the protein, which then results in a sudden increase of bead-to-bead distance…*"

The hypothetical scenario described by the reviewer that the observed unfolding defects may arise from altered substrate recognition rather than from impaired mechanochemical coupling would be valid if a ClpX hexamer with some mutant subunits is challenged with a stable folded substrate with a short tag for

recognition. In this case, if ClpXP has difficulty applying force, then the recognition tag cannot enter the ClpX pore to interact with the pore-1-loop, binding will be weak and therefore the substrate will be released. However, this is not the case in our assay when ClpXP has translocated all the unfolded substrate and is stalled in front of GFP.

Also, our ClpXP Class-I conformation, without substrate, has the same structure (RMSD = 0.83 Å) as the one reported by Ghanbarpour *et al*. (2023), having the wild-type central coupler PDB 8E91 (See Fig. 10f). However, the original Fig. 10f does not show pore-1-loop, pore-2-loop, and RKH loops, which are involved in substrate recognition and binding. The new Fig. 12a, b includes now these motifs which are described in the caption.

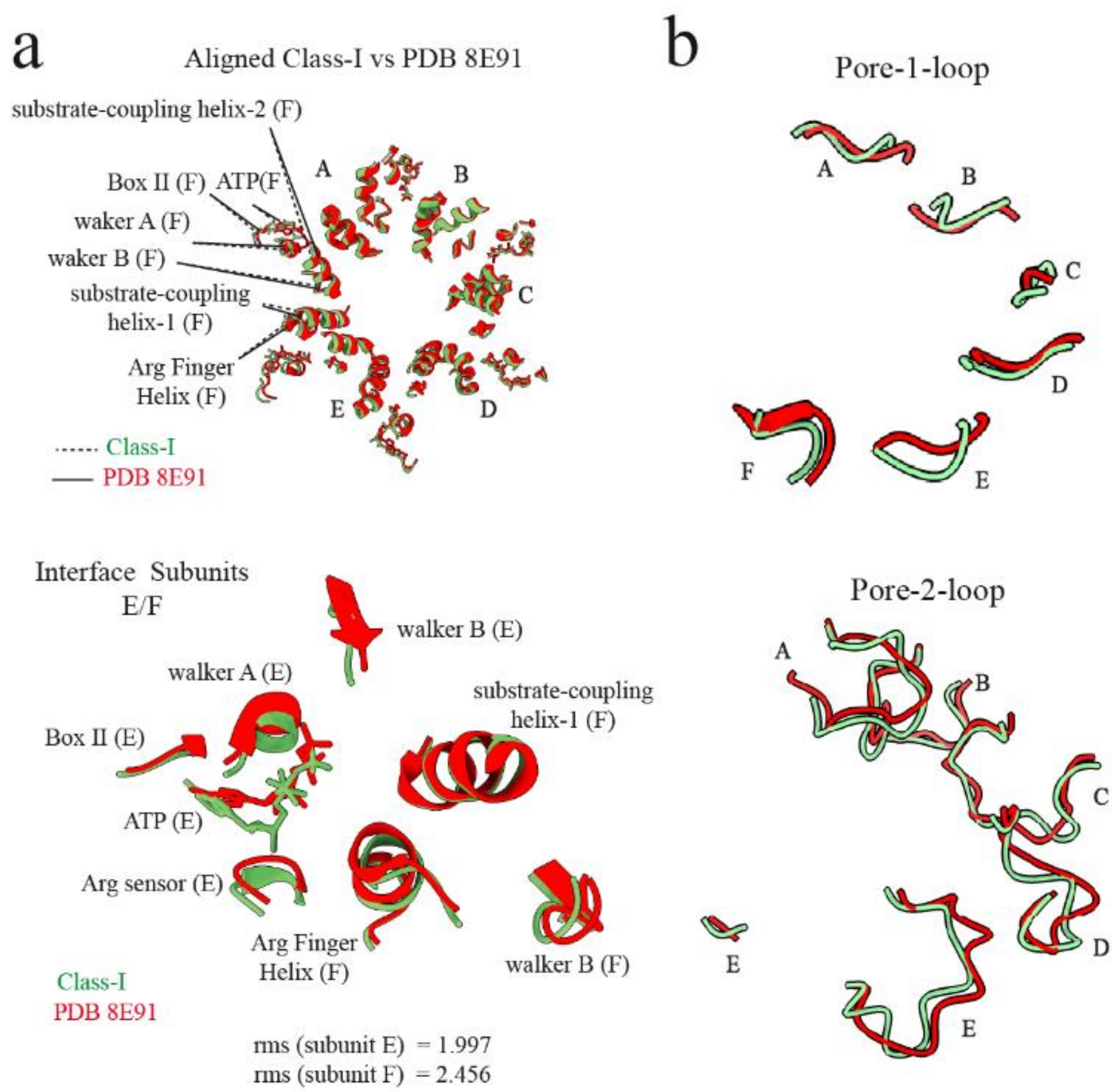

*"**Supplementary Fig. S12. Comparing cryo-EM ClpXP structures without substrate. a)** Overlapping of the ClpX coordinates of Class-I vs PDB 8E91. Bottom: close-up view of the overlapped structures in the Subunit E/Subunit F interface. The ClpX atomic model in Class-I (harboring the triple alternate mutation Q208A), closely matches the ClpX model in the substrate-free wild-type ClpXP structure PDB 8E91. **b)** Comparing the pore-1-loop (top) and pore-2-loop (bottom) of Class-I and PDB 8E91…"*

As can be seen, the pore-1-loop and pore-2-loop motifs of all subunits in Class-I and PDB 8E91 overlap well. This result provides another piece of evidence that substrate recognition is not affected by the

mutation of the central coupler since pore-1-loop and pore-2-loop are also involved in substrate binding. The RKH loops are not resolved in our structure due to their intrinsic flexibility.

We introduced this argument in lines 301 – 305 (changes are in red):

"*Overall, in the absence of a substrate, disruption of the central coupler in WMWMWM does not result in a structural change relative to wild-type ClpX. Furthermore, pore-1-loops and pore-2-loops, known to be involved in substrate recognition*[12]*, are seen in Class-I to adopt the same conformation as in PDB 8E91, suggesting that substrate recognition is not affected by Q208A.*"

4. The claim that Class II represents a new substrate-engaged stalled conformation requires more rigorous comparison with recently reported ClpX/ClpXP structures, including quantitative RMSD analyses. The current comparisons appear largely limited to Fei et al.'s ClpX structures, some of which were solved with Walker B mutations or with uncharacterized substrates. The authors should more clearly articulate how Class II differs structurally from previously described pre–power-stroke states (e.g., Ghanbarpour et al., Nature Communications, 2024). Additionally, how do the authors exclude the possibility that Class II reflects a mutation-stabilized off-pathway state rather than a physiologically relevant intermediate? It is notable that a comparable conformation has not been observed in wild-type ClpXP structures stalled with unfolding-resistant substrates (e.g., Ghanbarpour et al., Nature Communications, 2024). Addressing this discrepancy would be important to support the proposed mechanistic interpretation.

Answer. We thank the reviewer for pointing out the need to better support our claim that Class-II is a new pre-power stroke conformation. In this regard, we included other references and comparisons with more cryo-EM structures in several sections of the main text. We also added new supplementary Figures and table to illustrate this point. Lines 275 – 280 now read as follows:

"*After performing single-particle analysis, a total of three major ClpXP structural classes were obtained (Fig. 5a-c, Supplementary Data 1, Supplementary Table 2, 3). As reported in previous cryo-EM studies*[6,7,28–32]*, the ClpX subunits in the three classes the ClpX subunits in the three classes adopt a clockwise right-handed helical or spiral conformation ($A^W B^M C^W D^M E^W F^M$ from top to bottom, with the 'seam' between subunit F and A) that sits on top of one of the two ClpP homo-heptameric rings, contacting it via the IGF loops (Fig. 5a-c).*"

We also provide RMSD values for relevant comparison between ClpX structures in lines 295 – 297 (changes are in red):

"*Class-I resembles the substrate-free ClpXP reported PDB 8E91* [28] *(RMSD = 0.83 Å, Supplementary Fig. 12a). As in that study, we find that pore-2 loop of the subunit A^W appears in an 'extended' conformation…*"

Also, in lines 307 – 309 (changes are in red):

"*From all the previous cryo-EM studies*[6,7,28–32]*, Class-III closely resembles (RMSD = 1.92 Å) the substrate-bound ClpXP complex harboring a wild-type central coupler of PDB 6PP7*[7] *(Fig. 5d, Supplementary Table 4).*"

Lines 341 – 349 now includes the following modifications (changes are in red):

"Also, unlike our Class-II, in which the pore-1-loop is in an extended conformation towards the substrate, in other conformations, such as PDBs 6PP5, 6PP6 and 6PP8 [7], 9C87, 9C88, and 8V9R[29], and 6VFS and 6VFX[30], the pore-1-loop is not extended, but the entire subunit A is displaced closer to the substrate to interact with it. Moreover, Class-II also differs in the nucleotide states found in the mentioned structures[7,29,30] (Supplementary Table 4). Taken together, these observations indicate that, while Class-III resembles PDB 6PP7[7], Class-II differs significantly from previously reported cryo-EM ClpXP structures with substrate (Supplementary Table 4), and therefore it represents a new conformational state (see discussion below)."

We now include more comparisons with other pre-power stroke conformations in lines 374 – 387 (changes are in red):

"…Second, PDB 6VFS, 6VFX[30], 6PP5, 6PP6 and 6PP8[7] are post-power stroke conformations because the bottom subunit F has ADP (Supplementary Table 4) resulting from the transfer from subunit A to that position upon ATP hydrolysis and displaying the pore-1-loop nearby to the substrate. Also, in the same structures, the subunit at the top position A is loaded with ATP having the pore-1-loop closer to the substrate but unable to do the power stroke. Third, PDB 6PP7[7] represents a pre-power stroke state because subunits in position A and F are loaded with ADP and ATP, respectively, and the pore-1-loop in subunit A is not interacting with the substrate. Fourth, our Class-II has ADP and ATP bound to subunits A and F, respectively, like PDB 6PP7, but with the pore-1-loop of subunit A extended to interact with the polypeptide substrate. We interpret this structure as the transient intermediate between PDB 6PP7 and 6PP5 (Fig. 6b). Finally, the recent cryo-EM structures PDB 8V9R, 9C88 and 9C87[29] are a combination of pre- and post-power stroke states because both top and bottom subunits of the spiral have ADP, and the pore-1-loop of the top subunit is not interacting with the substrate, whereas the bottom subunit shows this interaction."

The new Supplementary Table 4 in the supplementary information is:

Supplementary Table 4: Class-II and Class-III vs previous cryo-EM structures with substrate within the ClpX pore

Ref.	PDB	Nucleotide*	State**	Walker-B mutation/ ATPγS	Force affected? ***	Top subunit to polypeptide⁺	Top Pore-1-loop to polypeptide⁺⁺
This study	9ORD	DTTTTT	Pre	No/Yes	Yes	Away	Closer
This study	9OTX	DTTTTT	Pre	No/No	Yes	Away	Away
Ghanbarpour *et al.*,[29]	8V9R	DTTTDD	Pre/post	No/No	No	Closer	Midway
Ghanbarpour *et al.*,[29]	9C87	DTTTDD	Pre/post	No/No	No	Closer	Midway
Ghanbarpour *et al.*,[29]	9C88	DTTTDD	Pre/post	No/No	No	Closer	Midway
Fei *et al.*,[46]	6PP8	TTTTTD	Post	Yes/Yes	No	Closer	Closer
Fei *et al.*,[46]	6PP7	DTTTTT	Pre	Yes/Yes	No	Away	Away
Fei *et al.*,[46]	6PP6	TTTTTD	post	Yes/Yes	No	Closer	Closer
Fei *et al.*,[46]	6PP5	TTTTTD	post	Yes/Yes	No	Closer	Closer
Ripstein *et al.*,[30]	6VFS	TTTTDD	Post	Yes/No	No	Closer	Closer
Ripstein *et al.*,[30]	6VFX	TTTTTD	Post	Yes/No	No	Closer	Closer

* The nucleotide occupancy of subunits from top to bottom of the ClpX spiral. D, denotes ADP, whereas T is ATP.

** The state of the ClpX conformation relative to the power stroke according to the PA/LS model.

*** If the generation of force by ClpX is being affected somewhat and if it was experimentally measured.

[+] Full top subunit relative to the polypeptide substrate.

[++] Position of the pore-1 loop motif relative to the polypeptide substrate.

We also have addressed the point raised by the reviewer that Class II may reflect a mutation-stabilized off-pathway state rather than a physiologically relevant intermediate and why Class-II has not been observed in other ClpXP structures. We have now modified lines 388-401 to make this point clear (changes are in red):

"*We presume that the discovery of this new pre-power stroke conformation (Class-II) was enabled by the combination of (i) ATP hydrolysis inhibition via ATPγS, (ii) the hindering of force generation with Q208A in WMWMWM (Supplementary Table 4), and (iii) the presence of a mechanically stable substrate in front of the motor, making Class-II long-lived enough to be captured under cryo-EM. Indeed, without ATPγS, we did not observe Class-II in our cryo-EM analysis with Q208A because Class-II, although present, may still be too short-lived. This was also the case of previous structures hindering only ATP hydrolysis with the Walker-B mutation and/or in the presence of ATPγS[7,30], or only challenging ClpX with an unfolding-resistant substrate[29]. Furthermore, WMWMWM is extremely sensitive to strong opposing force and high mechanical barriers (e.g. protein unfolding) but displays similar activity than WWWWWW in the absence of an external opposing force or when the barrier is minimal (translocation of unfolded polypeptides). These observations provide further support that the new conformation (Class-II) is on-pathway to the mechanism leading to the power stroke (Fig. 6b) and is only long-lived by the combination of the three conditions mentioned above.*"

Furthermore, we incorporate the new recent structures PDB 8V9R, 9C88 and 9C87 in the mechanism of action of ClpXP as the intermediate state between the extended and detached conformation of the pore-1-loop in Fig. 6b. Importantly, we included the relevant motifs of PDB 9C88 (as a representative of the structures mentioned above) in the molecular model shown in Fig. 6b:

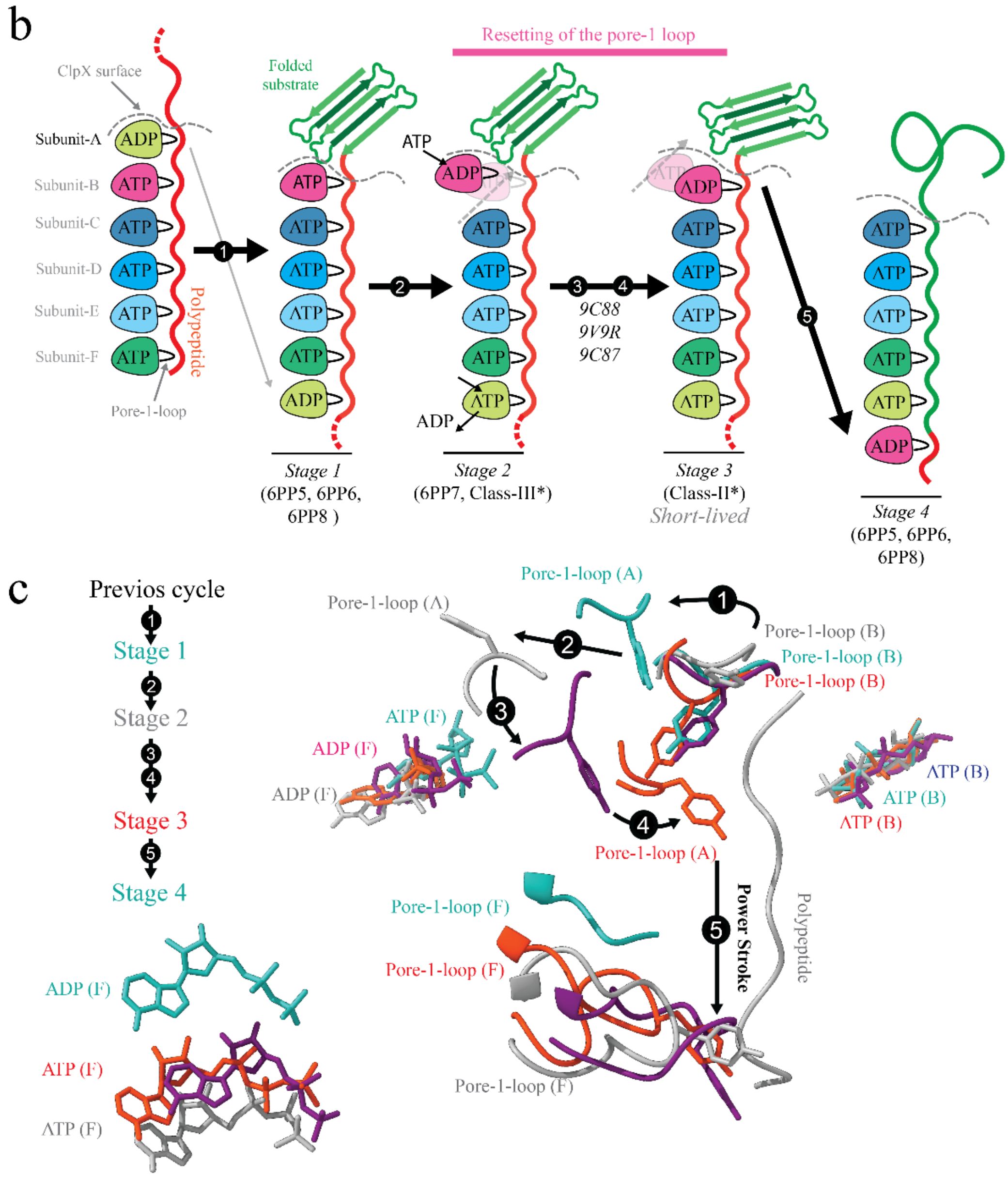

The caption of Fig. 6 has been modified accordingly:

"***b)*** *Schematic of the conformational states based on the cryo-EM structures observed previously*[7] *and those described here. An additional state is observed between PDB 6PP5, 6PP6 6PP8*[7]*, Class-III* (stage 4) and 6PP7*[7] *(stage 2) in the PA/LS model proposed by Sauer*[7]*. This intermediate, Class-II* (stage 3), is usually short-lived. *Refers to the coordinated version of Class-II and -III. Each subunit of the spiral (ABCDEF, from top to bottom) is represented with different colors. The folded protein (green color) is pulled down (power-stroke) by the downstream movement of top subunit A to bottom subunit F. ATP binding, hydrolysis (discontinuous arrows), and ADP release are annotated per ClpXP conformation. Between stage 3 and 4, the most recent cryo-EM structures (PDB 9C88, 9C87, 8V9R*[29]*) have a pore-1-loop in an intermediate conformation between its detached and extended states.* ***c)*** *Model of structural mechanism of the four conformational stages of the pore-1-loop to carry out the power stroke when the force generating subunit*

goes from position B to A and F in the spiral. Nucleotide status of subunit A, B and F of each structure are represented as sticks. Green = PDB 6PP5, Red = Class-II, Gray = Class-III, Purple = PDB 9C88."

Finally, we also included the new Supplementary Fig. 13 to show the similarity of previous structures among themselves.

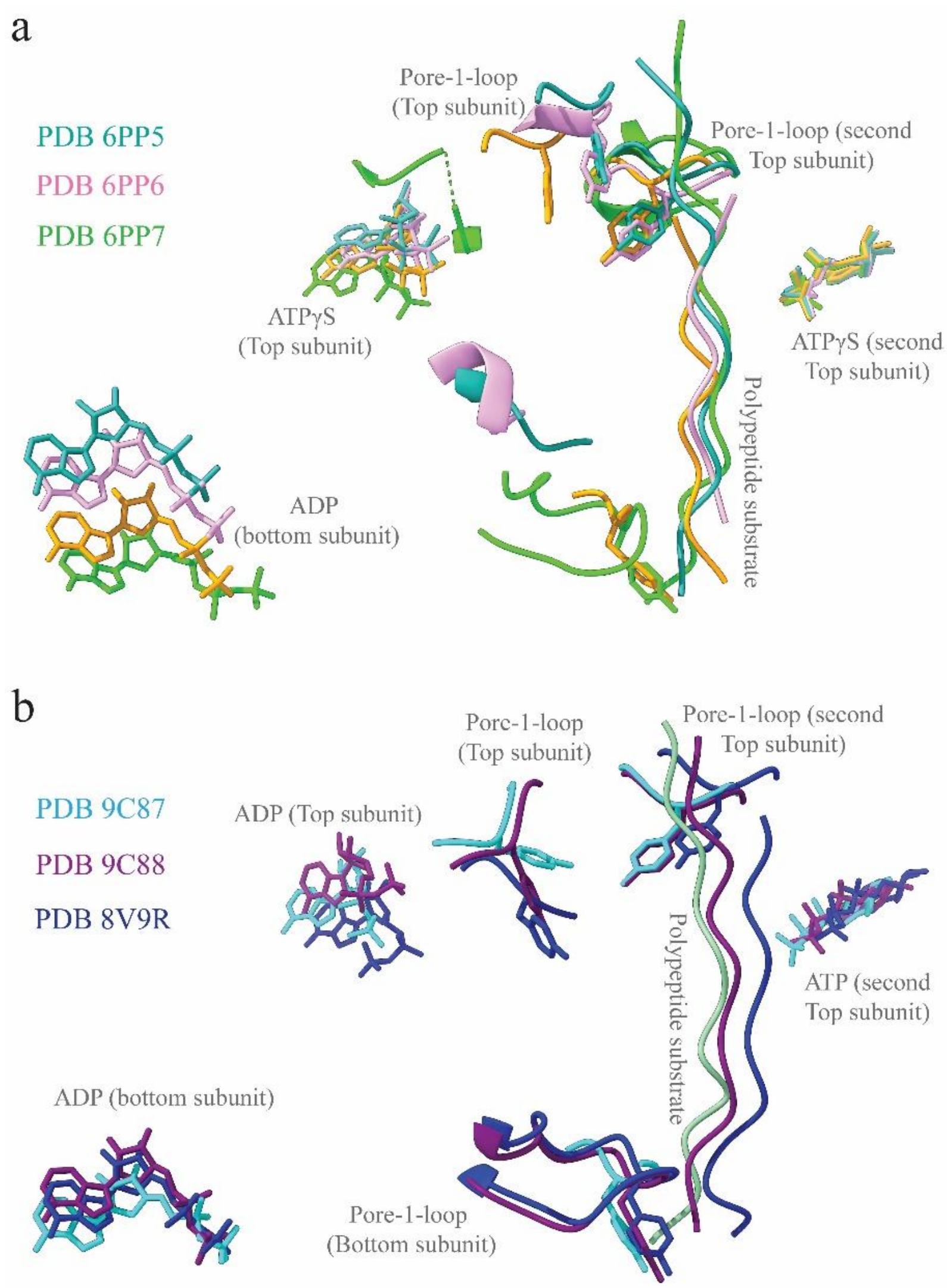

The caption of this new Supplementary Figures is:

*"**Supplementary Figure 13. Comparing the top, second top, and bottom subunits of other cryo-EM ClpXP structures with substrate. a)** for Fei et al., 2020[46] and **b)** Ghanbarpour et al., 2025[29]. Each PDB structure is depicted in different colors."*

5. The authors should clarify whether the cryo-EM density is sufficient to confidently assign which portion of the substrate is engaged or being translocated. Given the mechanistic emphasis on an "engaged" or pre-power stroke state, it is important to determine whether the map resolution and density continuity allow assignment of specific substrate segments within the axial channel. If the

density does not permit unambiguous modeling of the substrate, the conclusions regarding the precise engagement state should be appropriately toned down, as these particles could alternatively represent translocation or degradation complexes.

Answer. We thank the reviewer for raising this important point. In the text we used the words "engaged" or "dis-engaged" when the pore-1-loop is close or far away from the polypeptide substrate, rather than using the term "engaged" in the context of the ClpXP complex interacting with the substrate. To avoid confusion, in the revised version of the manuscript we now clarify this point by arguing that closeness of the pore-1-loop represents a conformation where the motor is applying force, in contrast to the case in which this loop is seen far from the substrate. Specifically, the text has been modified as follow in lines 406 – 419 (changes are in red):

"*Stage 2: the first of two ATP hydrolyses has taken place, the top subunit is found bound to ADP, and its pore-1-loop is away ("detached" conformation) from the substrate (PDB 6PP7[7], and our Class-III). Stage 3: the previous ADP has been ejected, another ATP has been bound, and a second hydrolysis has taken place leading to the extended conformation of the pore-1-loop to interact with the substrate (our Class-II), followed by the power stroke (involving the transition of the subunit from position A to position F). In the most recent cryo-EM structures (PDB 9C88, 8V9R, 9C87[29], Supplementary Fig. 13), the pore-1-loop of the top subunit, occupied with ADP, is located at an intermediate distance between the extended and detached conformations. Thus, these structures likely represent pre-power stroke states with the pore-1-loop located mid-way to interact with the polypeptide substrate (cyan color in Fig. 6c). It is possible that this conformation is observed when the motor faces an unfolding-resistant substrate. Finally, Stage 4 or post-power stroke, when subunit F is loaded with ADP, located at the bottom of the spiral, and its pore-1-loop remains near (presumably interacting with) the substrate (PDB 6PP5, 6PP6 and 6PP8[7]).*"

We also made modifications in lines 420 – 423 (changes are in red):

"*The extended and detached conformations of the pore-1-loop in the top subunit A observed in the present study, likely reflect the process of "resetting" that has been proposed for this loop [14,40] (Fig. 6b). We propose that before an ATP hydrolysis is used during the power stroke, a previous ATP hydrolysis is required for this resetting ~~of the pore-1-loop from the "detached" to the disengaged to engaged state~~. This model explains…*"

Also, the first two lines 456 – 457 of the last paragraph are (changes are in red):

"*We observe a new pre-power stroke conformation in which the pore-1-loop is ~~re-engaged and~~ ready to generate force…*"

We also modified Fig. 5a-c to rename the conformations of the pore-1-loop in Class-II and Class-III as extended and detached instead of engaged or dis-engaged.

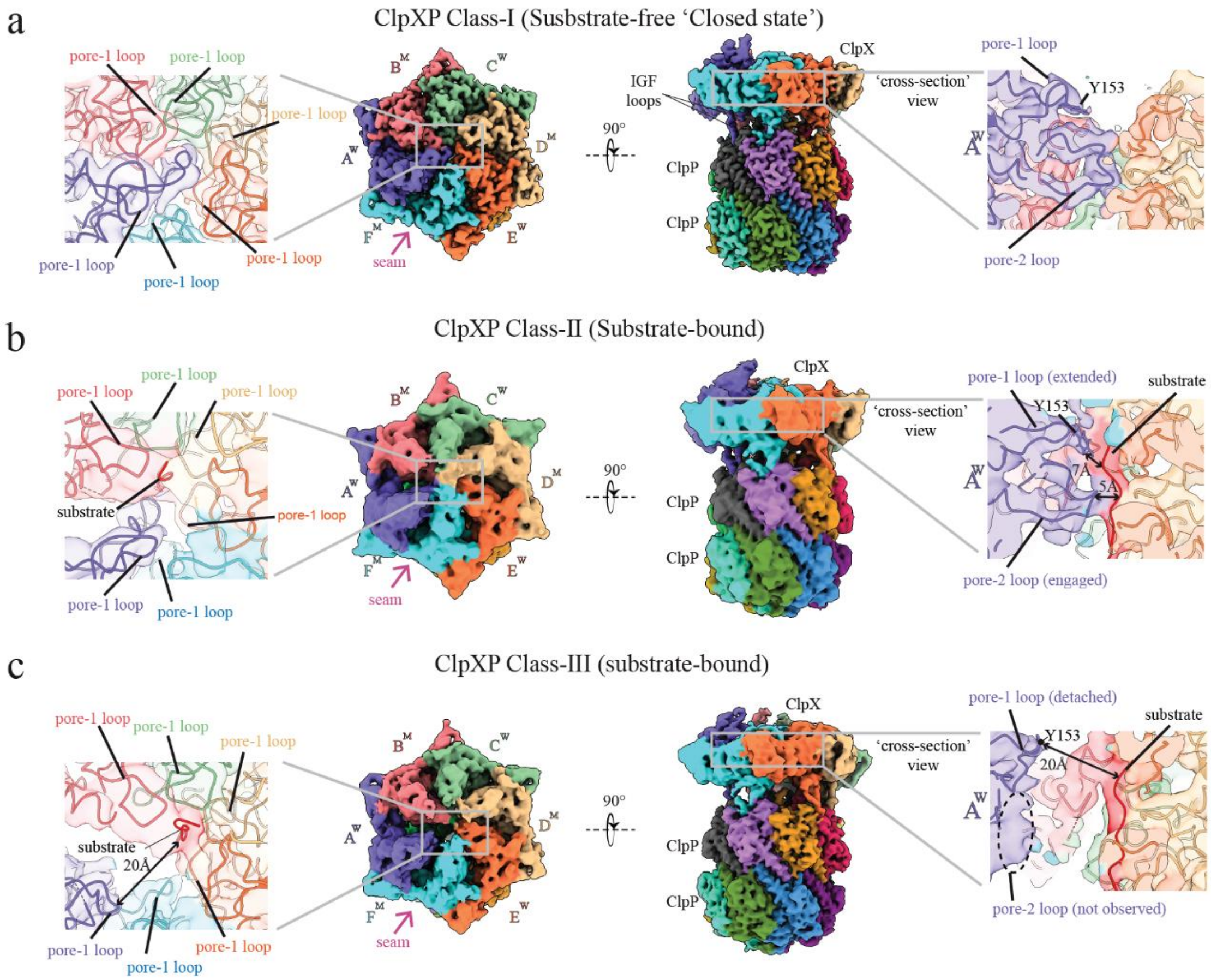

Also, to address the other point raised by the reviewer, the text has now been modified in lines 287 – 294 to indicate that we cannot determine the sequence of aminoacids of the substrate.

"*…At different threshold levels, Class-II and Class-III display an extra density on top of ClpX (Supplementary Fig. 9b), indicating that the motor is stalled in front of a mechanical barrier of a folded or partially folded structure. Although we cannot determine the sequence of aminoacids of the polypeptide within the ClpX pore (modeled as poly-Alanine), it is clearly threaded completely to the bottom subunit of the spiral. This feature is also found in other cryo-EM structures where E. coli's ClpXP is reported to be stalled, presumably applying force, in front of a folded structure that can or cannot be modeled[7,29] (Supplementary Fig. 11).*"

In support of the observation of the Class II intermediary, we have also added the new Supplementary Fig. 11 (below) for illustrative purposes comparing our structures with other previous cryo-EM studies.

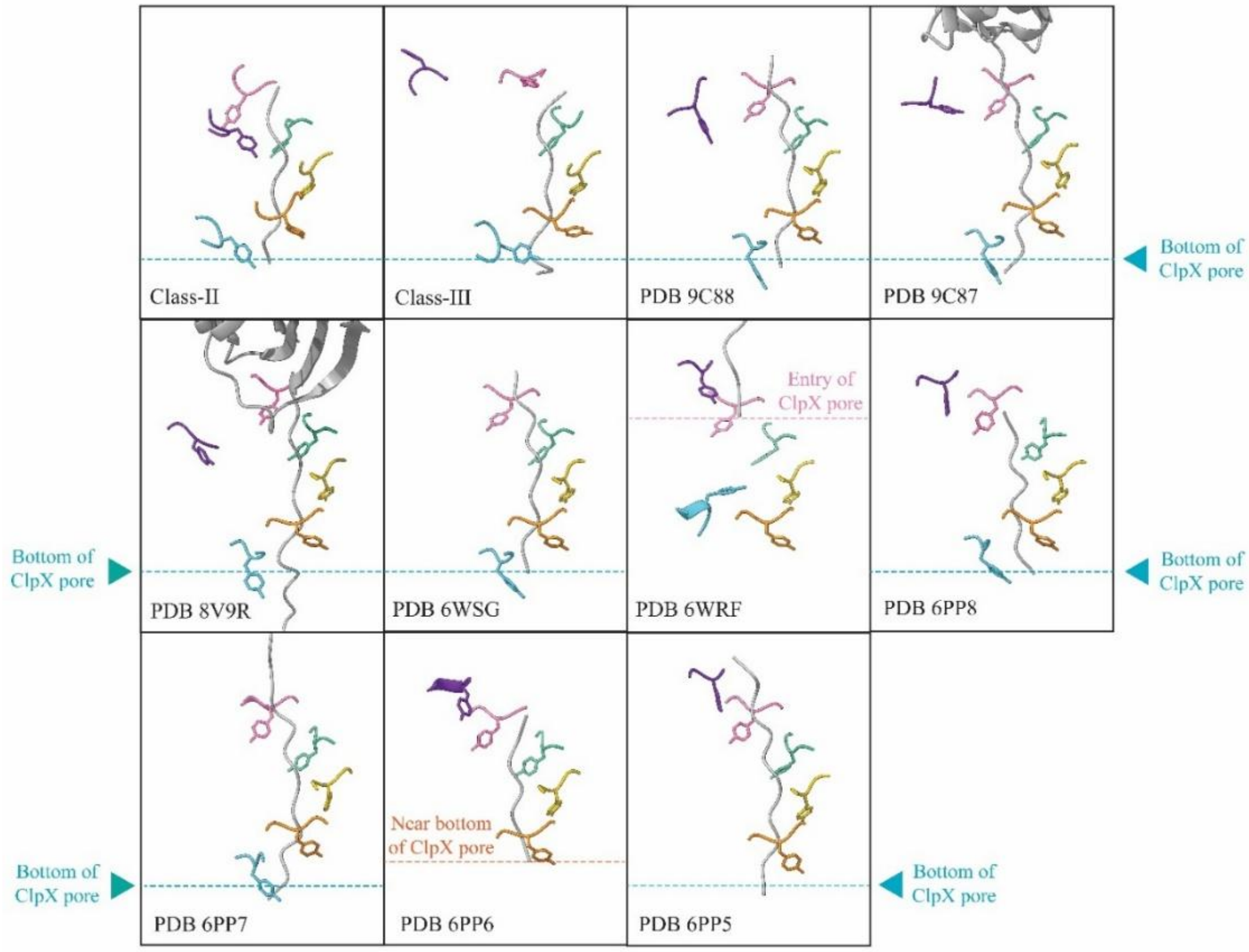

The caption read as follows:

"***Supplementary Figure 11. Comparing the substrate within the ClpX pore for different structures.*** *The PDB codes of the structures are indicated in each square. The polypeptide substrate within the ClpX pore is depicted in gray color. The pore-1-loop (aminoacids 152 to 153, with the Tyr153 in sticks) of each subunit (from bottom to top) are color-coded as in figure 5.*"

6. The Methods state that approximately one minute elapsed between the addition of ATPγS and grid freezing. Given the slow hydrolysis rate of ATPγS and the concentrations of ClpX, ClpP, and substrate used, it would be helpful for the authors to estimate the expected distribution of substrate-free, substrate-engaged, and translocation complexes under these conditions (e.g., Saunders et al., PNAS, 2020), and to assess whether the reported particle populations in each conformational state are consistent with the anticipated reaction conditions.

Answer: We thank the reviewer for the suggestion to estimate the relative populations of different ClpXP complexes. In this regard, however, we would like to clarify that such theoretical estimations of the distribution of different species (substrate-free, substrate-engaged, and translocation complexes) would not necessarily correlate with our experimentally observed structural results. This is due to specific challenges associated with the WMWMWM ClpX hexamer construct used in this study, and limitations in the single particle data analysis as detailed below. Notably, using the WMWMWM ClpX construct, our initial Heterogeneous Refinement results (Supplementary Data 1, right branch of the analysis) revealed that most of the particle population (from an initial subset of ~600,000 selected particles) corresponded

to ill-defined ClpXP complexes (~61%). These ill-defined particles comprised either completely free ClpP (~35 %) or particles in which the ClpX hexamer was poorly associated with ClpP (~26%). In contrast, only ~39% of particles displayed a well-defined ClpXP complex. These results indicate that, under cryo-EM conditions and in the presence of ATPγS, the WMWMWM ClpX construct exhibits limited efficiency in associating with ClpP to form stable and competent ClpXP complexes.

Furthermore, after 3D classification (without alignment) of the ~245,000 particles corresponding to well-formed ClpXP complexes, only approximately half of this subset refined to sufficiently high resolution to allow reliable structural interpretation. Among these, ~26% corresponded to a translocation complex, whereas ~35% represented a substrate-free population. The remaining 3D classes did not reach high resolution, precluding confident assessment of their conformational states. In addition, we would like to emphasize that quantitative estimation of population distributions from cryo-EM data inherently carries technical limitations. Particle classification outcomes can be influenced by preferred orientations, compositional and conformational heterogeneity, alignment bias, and differences in reconstruction quality between classes. Moreover, classes that fail to achieve sufficient resolution are typically excluded from detailed interpretation, which may further skew the apparent population distribution. Therefore, our cryo-EM–derived particle proportions have to be interpreted with caution and not be regarded as strictly quantitative measurements of solution-state equilibria.

7. The cryo-EM data-processing method section should provide more complete reporting. Please include classification strategy, masking procedures, map sharpening parameters, and any non-default software settings. Q-scores, directional resolution plot, angular distributions, and sphericity scores should also be reported for each structure.

Answer. We thank the reviewer for their comments and recommendations to strengthen our manuscript and expand on cryo-EM reported data-processing. We have added the new Supplementary Fig. 10 (below) containing the directional resolution plot, angular distributions, and sphericity scores; and the new Supplementary Table 3 (below) which contains the Q-scores calculated for each subunit. We have also modified the 'Cryo-EM data processing' section in our Methods (in red color) to detail our data analysis pipeline as requested. We have also modified the Supplementary Data 1 to detail every step followed in our pipeline. All model refinement and map sharpening parameters are also described in the Supplementary Table 1.

The new the Supplementary Fig. 10 with the caption is now:

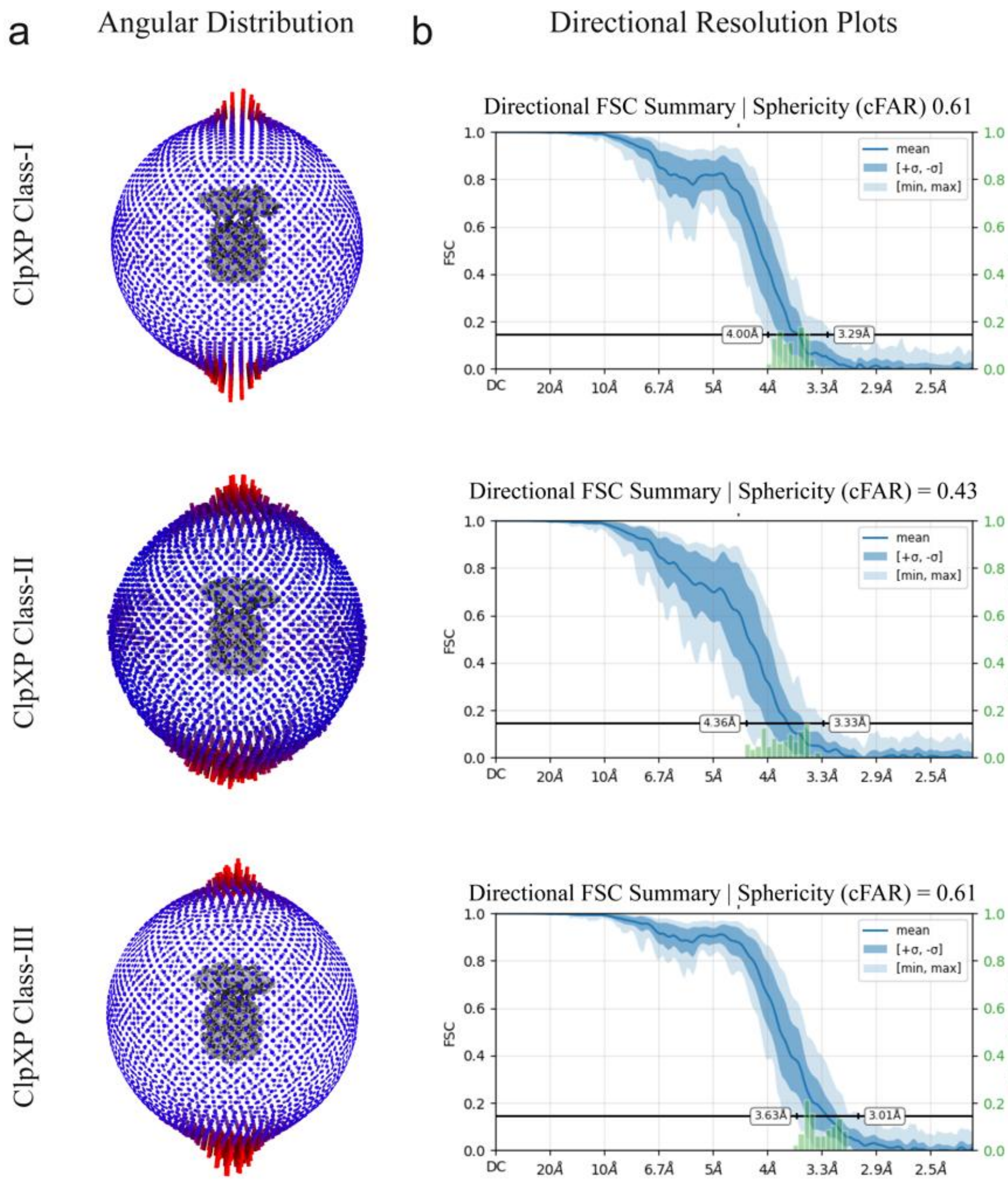

"***Supplementary Figure 10. Angular distribution and Directional Resolution Plots. a)*** *Angular distribution plots for the cryo-EM ClpXP class-I, -II and -III structures, as indicated.* ***b)*** *Directional Resolution FSC summary plots for the cryo-EM ClpXP class-I, -II and -III structures, as indicated. The reported sphericity score corresponds to the conical Fourier anisotropy ratio (cFAR).*"

And the new Supplementary Table 3.

Supplementary Table 3: Q-scores per subunit for the ClpXP Class-I, -II and -III structures

Chain	Q-score		
	**ClpXP class-I**	**ClpXP class-II**	**ClpXP class-III**
A	0.40	0.31	0.30
B	0.46	0.34	0.39
C	0.45	0.37	0.44
D	0.46	0.38	0.46
E	0.43	0.37	0.46
F	0.35	0.32	0.37

G	0.54	0.49	0.58
H	0.58	0.52	0.62
I	0.59	0.52	0.64
J	0.58	0.52	0.63
K	0.58	0.53	0.64
L	0.59	0.52	0.64
M	0.56	0.52	0.63
N	0.57	0.52	0.63
O	0.53	0.50	0.58
P	0.54	0.50	0.59
Q	0.54	0.50	0.58
R	0.54	0.50	0.58
S	0.54	0.50	0.57
T	0.55	0.49	0.58

"***13. Cryo-EM data processing***

The processing of the cryo-EM data was performed using cryoSPARC v4.4.1[47]. ~~All data analysis was performed using the cryosparc software v4.4.1~~. Movie frames were aligned using the Patch Motion Correction program and binned 2x (to 1.141 Å/pixel). Then, defocus estimation and the Contrast Transfer Function (CTF) fitting were performed using the Patch CTF Estimation program. In the corrected micrographs, we can readily observe particles that correspond to the ClpXP complex as well as to the free ClpP (Supplementary Fig. 9a). Data processing was conducted separately for conditions in which the complex was assembled with ATP (Data Set 1) or ATPγS (Data Set 2). A preliminary round of data analysis using a subset of each data set was performed, where the particles were picked using the blob-picker program to generate 2D classes that served for a follow template-based particle picking.

For Data Set 1, a subset of 5,861 particles was used to produce an ab initio 3D reconstruction. This volume was then used as a reference for a Heterogenous Refinement round (N=3) on ~700k particles selected from several rounds of 2D classification. From this Heterogenous Refinement round, one class of about 226k particles (~35%) displayed a stable ClpXP complex and was subjected to Homogenous refinement. Then, we performed a global 3D classification without alignment of this population, using only a solvent mask auto-generated on-the-fly. From this classification, one population of ~86k particles (~39%) was identified with the substrate inside the ClpX hexamer while another class of ~74k particles was observed to be in a substrate-free state. The population containing the substrate was selected, further cleaned to 76,882 particles and subjected then to non-uniform refinement. At high-threshold, this refined reconstruction showed a clear translocated substrate, while at low-thresholds a noisy ill-defined density appeared on top of the ClpX hexamer which was attributed to the non-translocated substrate region (Figure S9b). Then, a soft mask, involving only ClpXP including the translocated substrate within the ClpX hexamer, was used to perform particle subtraction followed by non-uniform refinement to finally produce the ClpXP class-III at 3.4 Å overall resolution (Supplementary Fig. 9, 10).

For Data Set 2, a similar workflow was performed (Supplementary Data 1). A subset of ~245k particles was subjected to Homogeneous Refinement followed by a 3D classification without alignment. Here, two

classes, accounting for ~26% (~63k particles) and ~35% (~59k particles) of the population, showed the best structural features, and were refined independently. The first class mentioned above (~63k particles), was selected and its population further cleaned to ~53k particles then subjected to non-uniform refinement. At high-threshold, this refined reconstruction showed a clear translocated substrate, while at low-thresholds a noisy ill-defined density appeared on top of the ClpX hexamer which was attributed to the non-translocated substrate region (Supplementary Fig. 9b). Then, a soft mask, involving only ClpXP with the translocated substrate within the ClpX hexamer, was used to perform particle subtraction followed by non-uniform refinement to finally produce the ClpXP class-II structure at 3.8 Å overall resolution (Supplementary Fig. 9, 10).

As mentioned before, in both data sets a particular 3D class corresponding to the substrate-free ClpXP complex was identified (denoted here as $^{(A)}$ and $^{(B)}$Substrate-free ClpXP complexes in Data S1). The particles were merged and further cleaned, resulting in ~68k particles producing the ClpXP Class-I structure obtained at 3.7 Å global resolution (Supplementary Fig. 9, 10).

14. Model Building and Refinement

The final refined maps of ClpXP Class-I, -II and -III, were sharpened with B-factors of -85, -30 and -30, respectively (Supplementary Table 1), to prepare them for the initial model building. For Class-I, the initial model was obtained by rigid-body fitting the atomic coordinates of the substrate-free wild-type ClpXP complex, PDB 8E91[7], into the corresponding sharpened map using UCSF ChimeraX[48]. For Class-II and Class-III complexes, the starting coordinate model was also obtained from PDB 8E91[7], but the pore-1 and -2 loops for subunit A, as well as the substrate bound, were rebuilt manually. For each complex, the models were then iteratively rebuilt in COOT[49] and refined using the real space refinement program in PHENIX[50]. For Class-I, the final sharpened cryo-EM map was obtained using the DeepEMhancer sharpening program[51]. For Class-II and Class-III, the final sharpened maps were obtained using the LocScale Program[52]. All validation and refinement statistics are shown in Table S1."

Minor comments:

1. Line 109: Section 3, not Section 4, etc.

Answer. We fixed this typo

2. Line 218: "WWWWM" one monomer designation appears to be missing.

Answer. We fixed this typo.

Methods:

3. Section 1: "Nitrogen Liquid" should be "Liquid nitrogen."

Answer. This typo has now been fixed in several parts of the material and methods.

4. Section 1: "0.44 µm filter" please verify whether this should be 0.45 µm?

Answer. We fixed this type.

5. Section 3: “1 m DTT” should be 1 mM DTT.

Answer. We fixed this typo.

6. Section 5: “guanidium chloride” should be “guanidinium chloride.”

Answer. We fixed this typo.

7. Section 11: “preparated” should be corrected to “prepared.”

Answer. We fixed this typo.

8. “stored at -80oC” should be written as “stored at −80 °C.”

Answer. We fixed this typo in several sections of the material and methods.

RESPONSE TO REVIEWERS' COMMENTS

Reviewer #3 Comments

The authors have addressed most of my comments, and the manuscript has been substantially improved. However, there are still a few points that should be addressed before publication.

1. Supplementary Table S4 should be made more quantitative and clearly defined. The classifications "Away," "Midway," and "Closer" are not explained, making it difficult to understand how these assignments were made. The authors should specify the structural metric used for each comparison, including the atoms or residues measured, the structural alignment procedure, and the distance thresholds used for each classification. In particular, the column "Top subunit to polypeptide" is ambiguous, as a whole subunit does not have a unique distance to the substrate.

Answer: We thank the reviewer for pointing out the lack of definition and unclear classifications in Supplementary Table 4. The alignment of cryo-EM structures used for the model presented in Figure 6C is now clearly defined in the text. We deleted the column "Top subunit to polypeptide" of Supplementary Table 4 because as the reviewer mentioned, the whole subunit does not have a unique distance to the substrate. We also deleted the column "Top Pore-1-loop to polypeptide" in the same table because now it is defined and explained in the text during the elaboration of the model of the sequential stages of the pore-1-loop. Furthermore, we measured the distance of the oxygen atom of the hydroxyl group of the Tyr153 of the pore-1-loop to the closest residue of the substrate located within the ClpX pore, δ (Å). The magnitudes of δ are now clear and substantially different (the values are now included in Supplementary table 4). All the changes mentioned above are included in main text between lines 426 and 446.

"*Based on the cryo-EM ClpXP structures with substrate observed previously*[7,29,30] *and those described here (all aligned relative to the invariant subunits C, D, E and substrate from PDB 9C88, PDB 6PP5, Class-II and Class-III), we propose a model in which the pore-1-loop of the top subunit in the spiral adopts conformations in four sequential stages during the power stroke (Fig. 6b, c). Stage 1: after power-stroke by subunit A, subunit B (loaded with ATP) occupies the position that subunit A had at the top of the spiral; see PDB 6PP5 (also 6PP6 and 6PP8*[7]*, Supplementary Fig. 14). Stage 2: the first of two ATP hydrolyses has taken place, the top subunit is found bound to ADP, and its pore-1-loop is in a detached conformation (the oxygen of Tyr153 OH-group is a distance δ = 26.3 Å away from the α-carbon of its closest substrate residue located within the ClpX pore, see Supplementary Table 4 and Fig. 6c); see Class-III (and PDB 6PP7*[7]*). Stage 3: the previous ADP has been ejected, another ATP has been bound, and a second hydrolysis has taken place leading to the extended conformation of the pore-1-loop (δ = 5.4 Å closer to interact with the substrate; see our Class-II and Supplementary Table 4, Fig. 6c), followed by the power stroke. In the most recent cryo-EM structures (PDB 9C88, 8V9R, 9C87*[29]*, Supplementary Fig. 14), the pore-1-loop of the top subunit, occupied with ADP, is located at an intermediate distance between the extended (δ = 5.4 Å) and detached (δ = 26.3 Å) conformations. Thus, these structures are likely to represent pre-power stroke states with the pore-1-loop located mid-way (δ = 10.9 Å for 9C88, Supplementary Table 4, Fig. 6c) to interact with the polypeptide substrate (Fig. 6c). Finally,*

Stage 4 or post-power stroke, when subunit F is loaded with ADP, located at the bottom of the spiral, and its pore-1-loop remains near (presumably interacting with) the substrate; see PDB 6PP5 (also 6PP6 and 6PP8[7])…"

See modified Supplementary Table 4

Reference	PDB	Nucleotide*	State**	Walker-B mutation/ATPγS	Force affected***	δ (Å)$^{+}$
This study (Class-II)	9ORD	DTTTTT	Pre	No/Yes	Yes	5.4
This study (Class-III)	9OTX	DTTTTT	Pre	No/No	Yes	26.3
Ghanbarpour *et al.*,[2]	8V9R	DTTTDD	Pre/post	No/No	No	13.5
Ghanbarpour *et al.*,[2]	9C87	DTTTDD	Pre/post	No/No	No	8.5
Ghanbarpour *et al.*,[2]	9C88	DTTTDD	Pre/post	No/No	No	10.9
Fei *et al.*,[1]	6PP8	TTTTTD	Post	Yes/Yes	No	4.8
Fei *et al.*,[1]	6PP7	DTTTTT	Pre	Yes/Yes	No	N.D^{++}
Fei *et al.*,[1]	6PP6	TTTTTD	Post	Yes/Yes	No	N.D^{++}
Fei *et al.*,[1]	6PP5	TTTTTD	Post	Yes/Yes	No	N.D^{++}
Ripstein *et al.*,[3]	6VFS	TTTTDD	Post	Yes/No	No	10.6^{+++}
Ripstein *et al.*,[3]	6VFX	TTTTTD	Post	Yes/No	No	7.6

* The nucleotide occupancy of subunits from top to bottom of the ClpX ring spiral. D, denotes ADP, whereas T is ATP.

** The state of the ClpX conformation relative to the power stroke according to the PA/LS model.

*** If the generation of force by ClpX is being affected somewhat and if it was experimentally measured.

$^{+}$ The distance between the oxygen of Tyr153 OH-group from the α-carbon of its closest substrate residue located in the ClpX pore.

$^{++}$ Not determined because Tyr153 of top subunit (pre-power stroke) was not resolved in PDB 6PP7. Also, Tyr153 of bottom subunit (post-power stroke) was not resolved in PDB 6PP6 and PDB 6PP5.

$^{+++}$ This value seems away from the substrate, but the substrate was not solved all the way to the bottom of the ClpX pore.

2. The new experiments examining the Q208A mutation in monomeric ClpX should be incorporated into the manuscript. While it is understandable that a given mutation may have different effects in the native enzyme and the covalently linked single-chain hexamer, the latter was specifically designed to distinguish defects in oligomerization from defects in mechanochemical function. The comparison between monomeric and single-chain Q208A is therefore directly relevant to the current study and strengthens the rationale for using the single-chain hexamer as the experimental system.

Answer: We thank the reviewer for the suggestion. We now include the results related to the monomer mutant and modify the main text in a way to justify the use of single-chain constructs. All the changes are included in main text between lines 84 and 117.

"…*Simulations of the T4 DNA clamp loader*[20] *indicate that mutation Q118A eliminates H-bonds that link the walker B motif and the Arg-finger helix. Accordingly, we introduced the corresponding mutation (Q208A) in the ClpX monomer. Among other possible residues forming H-bonds in the central coupler of ClpX, Q208A disrupts the network of H-bonds (Fig. 1c), weakening its interaction with the Walker-B and Arg-finger helix, resulting in a flexible central coupler.*

ClpX monomer mutant fails to assemble into active hexamers

The mild mutation of the glutamine residue (Q118N) in the T4 DNA clamp loader suggest that it may disrupt hexamer assembly[20]. We tested whether Q208A ClpX monomers (mono^{QA}) could self-assemble into active hexamers. Wild-type monomers (mono^{WT}) self-assemble, resulting in a broad peak in the size exclusion chromatogram, with the earliest region corresponding to the hexamer form and later regions corresponding to lower-order oligomerization states (Supplementary Fig. 1). In contrast, the chromatogram of mono^{QA} showed no absorbance around the hexamer peak, indicating that mutant monomers are unable to form hexamers (Supplementary Fig. 1). However, single-chain constructs (MMMMMM and WWWWWW) displayed a similar peak as the mono^{WT} hexamer (Supplementary Fig. 1). Consistently, a degradation assay of Titin^{CM}-ssrA using SDS-PAGE revealed that hexamers formed by mono^{WT} and WWWWWW were active, while MMMMMM was active but significantly slower, and mono^{QA} showed no activity (Supplementary Fig. 1, inset). Therefore, the disruption of the H-bonded junction in ClpX monomers results in their inability to self-assemble into active hexamers. To distinguish defects in oligomerization from ClpX functional activities, we studied the single-chain ClpX mutant constructs (Fig. 1d).

Single-chain ClpX constructs

We introduced the Q208A mutation in the covalently-linked subunits of single-chain ClpX hexamers[21]. This latter architecture allows us to (i) precisely control the number and spatial positioning of Q208A subunits (Fig. 1d) and (ii) study processes related to the mechanochemical cycle (ATP hydrolysis, coupling and force generation) and downstream functional activities (polypeptide translocation and protein unfolding) of fully assembled ClpX hexamer without oligomerization defects. In these constructs we refer to subunits as either "W" (for wild-type) and "M" (for subunit harboring the mutation Q208A). For example, WWWWWM and WWWMMM refer to constructs with a single-isolated mutant or occupying the three contiguous positions in the single-chain hexamer, respectively. WWWWWW, WWWWWM, WWWMWM and WMWMWM reveal the effect of adjacent wild-type and mutant subunits relative to two adjacent mutant subunits in WWWWMM, WWWMMM, MWWMMM (Fig. 1d). Comparing the activity of WWWMMM vs WMWMWM provides the most contrasting effect of the configuration of mutant subunits…"

We also added the following at the end of the section "Cryo-EM structure of WMWMWM ClpXP complexes":

"*Altogether, the fact that we can obtain fully assemble ClpX hexamers and that Class-I (no substrate) is identical to a ClpXP complex with the wild-type central coupler also demonstrate that the single-chain construct allows full formation of the ring without oligomerization defects.*"
