# Supplementary material for: The central coupler of ClpXP controls intersubunit communication and couples intrasubunit chemical energy with force generation
Source: Nat Commun. 2026 Oct 12;17:11580. doi: 10.1038/s41467-026-78225-1 (PMC13752256; doi:10.1038/s41467-026-78225-1)
Supplement: Supplementary file 3 [file 41467_2026_78225_MOESM3_ESM.pdf]

nature portfolio

Corresponding author(s): NCOMMS-25-93339A

Last updated by author(s): Apr 29, 2026

Reporting Summary

Nature Portfolio wishes to improve the reproducibility of the work that we publish. This form provides structure for consistency and transparency in reporting. For further information on Nature Portfolio policies, see our Editorial Policies and the Editorial Policy Checklist.

Statistics

For all statistical analyses, confirm that the following items are present in the figure legend, table legend, main text, or Methods section.

n/a	Confirmed	
☐	☒	The exact sample size (n) for each experimental group/condition, given as a discrete number and unit of measurement
☐	☒	A statement on whether measurements were taken from distinct samples or whether the same sample was measured repeatedly
☐	☒	The statistical test(s) used AND whether they are one- or two-sided<br>*Only common tests should be described solely by name; describe more complex techniques in the Methods section.*
☒	☐	A description of all covariates tested
☐	☒	A description of any assumptions or corrections, such as tests of normality and adjustment for multiple comparisons
☐	☒	A full description of the statistical parameters including central tendency (e.g. means) or other basic estimates (e.g. regression coefficient) AND variation (e.g. standard deviation) or associated estimates of uncertainty (e.g. confidence intervals)
☐	☒	For null hypothesis testing, the test statistic (e.g. F, t, r) with confidence intervals, effect sizes, degrees of freedom and P value noted<br>*Give P values as exact values whenever suitable.*
☒	☐	For Bayesian analysis, information on the choice of priors and Markov chain Monte Carlo settings
☒	☐	For hierarchical and complex designs, identification of the appropriate level for tests and full reporting of outcomes
☒	☐	Estimates of effect sizes (e.g. Cohen's d, Pearson's r), indicating how they were calculated

Our web collection on statistics for biologists contains articles on many of the points above.

Software and code

Policy information about availability of computer code

Data collection	The LabVIEW code is available at https://doi.org/10.5281/zenodo.4632595
Data analysis	the MATLAB code for analysis is available at https://zenodo.org/records/18810738.

For manuscripts utilizing custom algorithms or software that are central to the research but not yet described in published literature, software must be made available to editors and reviewers. We strongly encourage code deposition in a community repository (e.g. GitHub). See the Nature Portfolio guidelines for submitting code & software for further information.

Data

Policy information about availability of data

All manuscripts must include a data availability statement. This statement should provide the following information, where applicable:

- Accession codes, unique identifiers, or web links for publicly available datasets
- A description of any restrictions on data availability
- For clinical datasets or third party data, please ensure that the statement adheres to our policy

The single-molecule data collected in this study is available at https://zenodo.org/records/21879510

Research involving human participants, their data, or biological material

Policy information about studies with human participants or human data. See also policy information about sex, gender (identity/presentation), and sexual orientation and race, ethnicity and racism.

Reporting on sex and gender	This information has not been collected and it is not necessary in our study
Reporting on race, ethnicity, or other socially relevant groupings	This information has not been collected and it is not necessary in our study
Population characteristics	This information has not been collected and it is not necessary in our study
Recruitment	This information has not been collected and it is not necessary in our study
Ethics oversight	This information has not been collected and it is not necessary in our study

Note that full information on the approval of the study protocol must also be provided in the manuscript.

Field-specific reporting

Please select the one below that is the best fit for your research. If you are not sure, read the appropriate sections before making your selection.

- [x] Life sciences
- [ ] Behavioural & social sciences
- [ ] Ecological, evolutionary & environmental sciences

For a reference copy of the document with all sections, see nature.com/documents/nr-reporting-summary-flat.pdf

Life sciences study design

All studies must disclose on these points even when the disclosure is negative.

Sample size	No statistical method was used to predetermine sample size; instead, they were established based on previous publications and data collection was gathered until the standard error of the mean fell below 10% of the average. Cryo-EM sample size was determined by collecting data until the global resolution was below 4 Å.
Data exclusions	Predefined data exclusion criteria were applied to the single-molecule and cryo-EM datasets (all details are mentioned in the material and methods of each procedure)
Replication	All single-molecule and cryo-EM datasets were independently reproduced across multiple rounds of data collection
Randomization	To minimize potential systematic errors, data for different experimental conditions were interleaved and collected in a randomized order.
Blinding	Investigators were not blinded during experiments and outcome assessment. However, potential bias was mitigated by analyzing all data sets via an identical, automated, and predefined computational pipeline.

Reporting for specific materials, systems and methods

We require information from authors about some types of materials, experimental systems and methods used in many studies. Here, indicate whether each material, system or method listed is relevant to your study. If you are not sure if a list item applies to your research, read the appropriate section before selecting a response.

Materials & experimental systems

n/a	Involved in the study	
[x]	[ ]	Antibodies
[x]	[ ]	Eukaryotic cell lines
[x]	[ ]	Palaeontology and archaeology
[x]	[ ]	Animals and other organisms
[x]	[ ]	Clinical data
[x]	[ ]	Dual use research of concern
[x]	[ ]	Plants

Methods

n/a	Involved in the study	
[x]	[ ]	ChIP-seq
[x]	[ ]	Flow cytometry
[x]	[ ]	MRI-based neuroimaging

Plants

Seed stocks	This information has not been collected and it is not necessary in our study
Novel plant genotypes	This information has not been collected and it is not necessary in our study
Authentication	This information has not been collected and it is not necessary in our study
